# Real‐Life Workup of Chronic Hand Eczema Using a Dedicated Case Report Form: A SIDAPA Multicentre Study

**DOI:** 10.1111/cod.70105

**Published:** 2026-02-15

**Authors:** Rosella Gallo, Fabrizio Guarneri, Katharina Hansel, Luca Stingeni, Maddalena Napolitano, Cataldo Patruno, Monica Corazza, Alessandro Borghi, Ilaria Trave, Emanuela Martina, Maria Michela Lauriola, Rosanna Rita Satta, Caterina Foti, Paolo Romita, Roberta Giuffrida, Silvia Mariel Ferrucci, SIDAPA CHE Study Group, Gabriele Biondi, Gabriele Casciola, Florenzia Marielli, Roberto Pinto, Ilaria Salvi

**Affiliations:** ^1^ Section of Dermatology, Department of Health Sciences University of Genoa and Ospedale Policlinico San Martino, IRCCS Genoa Italy; ^2^ Section of Dermatology, Department of Clinical and Experimental Medicine University of Messina Messina Italy; ^3^ Dermatology Section, Department of Medicine and Surgery University of Perugia Perugia Italy; ^4^ Section of Dermatology, Department of Clinical Medicine and Surgery University of Naples Federico II Naples Italy; ^5^ Department of Medicine and Health Sciences Vincenzo Tiberio University of Molise Campobasso Italy; ^6^ Section of Dermatology and Infectious Diseases, Department of Medical Sciences University of Ferrara Ferrara Italy; ^7^ Section of Dermatology, Department of Health Sciences University of Genoa, IRCCS, Ospedale Policlinico San Martino Genoa Italy; ^8^ Dermatology Unit Ospedale Carlo Urbani Jesi Ancona Italy; ^9^ Istituti Ospedalieri Bergamaschi, Policlinico San Marco, Dermatology Unit Zingonia Osio Sotto Bergamo Italy; ^10^ Department of Medicine, Surgery and Pharmacy University of Sassari Sassari Italy; ^11^ Section of Dermatology, Department of Precision and Regenerative Medicine and Jonian Area University Aldo Moro of Bari Bari Italy; ^12^ Dermatology Unit Fondazione IRCCS Ca' Granda Ospedale Maggiore Policlinico Milan Italy

**Keywords:** atopic dermatitis, case report form, chronic hand eczema, contact dermatitis, diagnostic workup, patch test, quality of life, risk factors

## Abstract

**Background:**

Chronic hand eczema (CHE) is a challenging condition with multifactorial pathomechanisms and a wide clinical polymorphism. It is often resistant to treatments.

**Objectives:**

To clinically and etiologically investigate CHE patients using a case report form (CRF) developed for this purpose.

**Methods:**

A cross‐sectional study on adult patients affected by CHE was performed from January 2024 to May 2025 in 10 Italian dermatology clinics. Demographic data, clinical features, disease severity and duration, endogenous/environmental risk factors, patch test results, response to past/current treatments, and burden of disease were recorded in the dedicated CRF and analysed.

**Results:**

A total of 207 patients were enrolled in the study (mean age 41.1 ± 15.6 years), 142 (68.6%) females. CHE was moderate–severe in 58.9% of cases, refractory to topical potent corticosteroids in 81.4%. The etiological subtypes were irritant contact dermatitis in 52.7%, allergic contact dermatitis in 24.2%, and atopic dermatitis in 16.9%; clinical subtypes were identified in only 29.0% of patients, the most frequent ones being hyperkeratotic eczema (12.1%) and acute recurrent vesicular eczema (9.7%). The CRF proved to be easy to fill in and useful.

**Conclusions:**

An accurate clinical workup can lead to CHE clinical and etiological classification in about 80% of patients and may facilitate tailored treatment strategies.

## Graphical abstract

From January 2024 to May 2025, 207 patients (68.6% females) affected by CHE were enrolled to be clinically and etiologically investigated using a case report form specifically developed. The accurate workup led to clinical and etiological classification of CHE in about 80% of patients, allowing tailored treatment strategies.

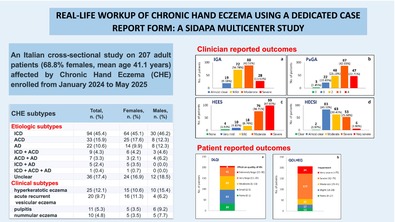

## 1 Introduction

Chronic hand eczema (CHE) is defined as eczema of the hands and wrists that persists for more than 3 months or recurs at least twice within a 12‐month period [1, 2, 3]. It is a common and challenging skin condition that significantly impacts daily activities and health‐related quality of life [4]. In up to one‐third of affected individuals, the disease is moderate to severe and results in a particularly highly reduced quality of life (QoL) and socioeconomic burden [5, 6].

CHE often has a multifactorial aetiology, where both endogenous and environmental factors can play a role [3]. On one side, an atopic background significantly increases susceptibility [1, 7, 8] on the other side, exposure to contact irritants and allergens is crucial in inducing and exacerbating the condition. CHE is most common in people who work in high‐risk occupations, such as wet work ones, characterised by a high cumulative exposure to these triggers [9]. It is approximately two times more frequent in women, probably due to higher exposure to irritants or allergens in both domestic and work environments [1, 2, 3, 4]. Other risk factors for CHE include prior history of hand eczema (HE), low age at onset of HE, having a contact allergy, cold/dry weather, and decreased indoor humidity [10].

Clinically, CHE may manifest with polymorphic features that include varying degrees of erythema, vesicles, oedema, papules, scaling, hyperkeratosis, lichenification, and fissures associated with itching, burning, and pain [1].

Due to this wide heterogeneity, the classification of CHE is challenging. Several classification approaches have been proposed but none is universally accepted. The European Society of Contact Dermatitis (ESCD) guidelines for hand eczema propose a mixed classification system incorporating both etiological and morphological subtypes [10]. Etiologically, irritant contact dermatitis (ICD), allergic contact dermatitis (ACD), hand‐localised atopic dermatitis (AD), and protein contact dermatitis are distinguished. Morphologically, acute recurrent vesicular, hyperkeratotic, nummular, and pulpitis subtypes are categorised. Unfortunately, the frequent coexistence of more than one subtype and the potential for diagnostic transitions over time makes this classification rigid and difficult to apply in real‐world clinical practice where CHE presents more as a spectrum of related conditions rather than a single disease entity [11].

Further complexity derives from the multifactorial pathogenesis of CHE, which involves disruption of the skin barrier, alterations of the skin microbiome, and dysregulation of inflammatory responses [1, 2, 3]. Each aetiology seems to be associated with unique immune signatures, suggesting that CHE classification, and therefore identification, could be improved by understanding the distinct immune signatures between subtypes, resulting in the possibility to target treatments [12, 13].

The management of CHE follows a stepwise approach from general care and barrier protection, associated with lifestyle changes and avoidance of triggering environmental factors, to topical and systemic therapies. Among systemic therapies, alitretinoin and off‐label immunosuppressants, like cyclosporine, are so far the only option to treat severe refractory cases. Newer drugs, such as interleukin (IL)‐4/IL‐13 inhibitors and JAK inhibitors, have shown promise for the treatment of CHE [14, 15, 16, 17, 18, 19, 20, 21]. Among the latter, the topical pan‐JAK inhibitor delgocitinib cream 20 mg/g was approved by the European Medicines Agency in September 2024 for moderate to severe CHE in adults for whom topical corticosteroids are inadequate or inappropriate.

The primary objective of this study is to systematically record and analyse the characteristics of patients affected by CHE, namely: demographic data, clinical presentation, disease severity and duration, risk factors, both endogenous and environmental, relevant environmental triggers (with particular attention to contact allergens), burden of disease, and response to past and current treatments. The secondary objective is to test the usefulness and ease of use of a specifically developed case report form (CRF), with a view to employing it as a tool for further prospective studies on CHE.

## 2 Materials and Methods

This is a cross‐sectional study designed and performed by SIDAPA (Italian Society of Allergological, Occupational and Environmental Dermatology) from January 2024 to May 2025 in 10 patch test clinics homogenously distributed in Italy. Adult patients affected by CHE were enrolled, excluding subjects with other inflammatory and infectious diseases involving the hands, such as psoriasis and mycoses. Eligible participants provided written informed consent for the collection of their clinical data and for data protection, in accordance with Good Clinical Practice (GCP) standards.

Each patient underwent detailed medical history including time of onset, disease course (episodic‐recurrent or persistent), demographic (sex, age, body height and weight, educational level) and occupational data, smoking habits, atopic and/or psoriatic family background, personal history or presence of AD and other atopic diseases, association between work and onset/worsening of HE, loss of working days because of HE, frequency of exposure to possible irritants and allergens at work, at home and during hobbies, with a particular focus on the frequency of handwashing. A specific question about taking care of small children was included. Past and present topical and systemic treatments were also recorded. Occupations were classified into three categories: wetwork jobs, non‐wetwork jobs at risk for CHE, and jobs not at risk for CHE.

With regard to clinical evaluation, morphology and severity of CHE were evaluated and recorded by means of six validated scoring systems to quantify signs, symptoms, and disease severity, namely HEES (Hand Eczema Extent Score) [22], HECSI (Hand ECzema Severity Index) [23], IGA‐CHE (Investigator's Global Assessment of Chronic Hand Eczema) [24], PaGA‐CHE (Patient's Global Assessment of Chronic Hand Eczema) [25], NRS (Numerical Rating Scale) score for pruritus [26], NRS score for pain [27]. Furthermore, photos of the palmar and dorsal aspects of the hands and wrists of every patient were taken. The presence of eczematous lesions on other body locations was also recorded.

All patients underwent patch testing that was performed using Haye's Test Chambers (Haye's Service, Alphen aan den Rijn, the Netherlands) on Soffix tape (Artsana, Grandate, Italy), with allergens provided by SmartPractice Europe (Greven, Germany). All patients were patch tested with the SIDAPA baseline series [28] and additional allergens and/or SIDAPA integrative series were added based on anamnestic data. Patch test readings were performed on day (D)2, D4, and D7; irritant and doubtful responses were recorded as negative results [29]. Positive results were recorded and their specific relevance for CHE was assessed considering the patient's history, exposure, and clinical course. If patch tests had already been performed in the past, their results were also recorded, together with information about the avoidance of already known relevant allergens.

Etiological subtypes were defined [10], including cases of doubtful/undetermined aetiology. Morphological subtypes were also identified and recorded [10]. Quality of life assessment was performed through completion of DLQI (Dermatology Life Quality Index) [30] and QOLHEQ (Quality Of Life in Hand Eczema Questionnaire) [31, 32]. The clinical examination, patch test reading and relevance interpretation, as well as the scoring of the six scoring systems, were performed by a dermatologist or, in some cases, by a resident under the supervision of a senior dermatologist. All these data were recorded in a specifically designed CRF (Table S1) and statistically analysed. The CRF was filled in two steps, at first presentation and at completion of patch testing. It was filled in on paper, and the data were then transferred in an online electronic format, as a stand‐alone solution independent from local information systems.

Finally, the usefulness and ease of use of the CRF was assessed by means of a short questionnaire administered to all participating centres (Table S2).

Statistical analysis was performed summarising continuous data by mean and standard deviation, categorical data by frequency tables, minimum and maximum values and quartiles. Comparisons between subgroups of patients were made using Student's *t*‐test (if the required conditions were met) or Mann–Whitney's test for numeric data, while chi‐square test or Fisher's exact test, as appropriate, were used for categorical data. Benjamini‐Hochberg's correction for multiple comparisons, with a false discovery rate of 0.05, was used to define the significance threshold for *p* values. Intraclass correlation coefficient (ICC) was calculated to assess inter‐rater agreement on the opinions about the usefulness and ease of use of the CRF.

## 3 Results

In total, 207 adult patients with CHE were enrolled in the study, 142 of them were females (68.6%). The mean age was 41.1 ± 15.5 years (range 18–82). Females, on average, were younger than males (39.4 ± 15.0 vs. 44.8 ± 16.2 years, *p* = 0.032, not significant after Benjamini‐Hochberg's correction). Most patients had a lower educational degree (primary/secondary school, 72.0%), and this proportion was significantly different between males (84.6%) and females (66.2%) (*p =* 0.006) (Table 1).

**TABLE 1:** Baseline characteristics of the study population with chronic hand eczema.

	Total *n* = 207	Females *n* = 142 (68.6%)	Males *n* = 65 (31.4%)
Age (years), mean ± SD	41.1 ± 15.6	39.4 ± 15.0	44.8 ± 16.2
Age at CHE onset, mean ± SD	35.0 ± 17	33.4 ± 15.3	40.7 ± 18.3
Years from CHE onset, mean ± SD	6.0 ± 8.1	6.5 ± 8.9	4.8 ± 6.0
Instruction level, *n*. (%)			
Primary/secondary	149 (72.0)	94 (66.2)	55 (84.6)
Tertiary	58 (28.0)	48 (33.8)	10 (15.4)
BMI, mean ± SD	25.15 ± 4.6	24.5 ± 4.4	26.6 ± 4.8
Smoking habit, *n*. (%)			
Current smoker	54 (26.1)	34 (23.9)	20 (30.8)
Past smoker	24 (11.6)	12 (8.5)	12 (18.5)
Never smoked	129 (62.3)	96 (67.6)	33 (50.8)
Family history of atopy, *n*. (%)			
Yes	64 (30.9)	50 (35.2)	14 (21.5)
Uncertain	8 (3.9)	6 (4.2)	2 (3.1)
Personal history of atopic dermatitis, *n*. (%)			
Currently affected	39 (18.8)	30 (21.1)	9 (13.8)
Previously affected	25 (12.1)	20 (14.1)	5 (7.7)
Uncertain	11 (5.3)	6 (4.2)	5 (7.7)
Personal history of allergic rhinoconjunctivitis, *n*. (%)			
Currently affected	55 (26.5)	39 (27.5)	16 (24.6)
Previously affected	21 (10.1)	17 (12.0)	4 (6.2)
Uncertain	3 (1.4)	2 (1.4)	1 (1.5)
Personal history of allergic asthma, *n*. (%)			
Currently affected	21 (10.1)	15 (10.6)	6 (9.2)
Previously affected	13 (6.3)	9 (6.3)	4 (6.2)
Uncertain	3 (1.4)	1 (0.7)	2 (3.1)
Known sensitization to allergens, *n*. (%)			
Aeroallergens	74 (35.8)	50 (35.2)	24 (36.9)
Food allergens	13 (6.3)	9 (6.3)	4 (6.2)
Hymenoptera venom/drugs	9 (4.3)	7 (4.9)	2 (3.1)
Family history of psoriasis, *n*. (%)			
Yes	32 (15.5)	25 (17.6)	7 (10.8)
Uncertain	5 (2.4)	2 (1.4)	3 (4.6)
Personal history of psoriasis, *n*. (%)			
Yes	6 (2.9)	3 (2.1)	3 (4.6)
Uncertain	9 (4.4)	4 (2.8)	5 (7.7)

Abbreviations: BMI, body mass index; CHE, chronic hand eczema; SD, standard deviation.

Age of onset of CHE was 35.7 ± 16.6 years (range 1–82) and was significantly lower in females than in males (33.4 ± 15.3 vs. 40.7 ± 18.3 years, *p =* 0.006). Only in 11 patients, the age of onset was < 16 years; all these patients were atopic (10 with past or current AD). The mean disease duration was 6.0 ± 8.1 years (range < 1 to 44), without significant difference between males and females (*p =* 0.95).

Current AD was documented in 56/207 (27.1%) patients, of which 42 females (29.6%) and 14 males (21.5%); in 39 cases the disease had been previously identified, while in the remaining 17 it was diagnosed at the enrolment visit. AD was reported only in childhood by 8.7% (18/207; 13 females, 9.2%; 5 males, 7.7%). Respiratory or food allergies without AD were reported by 55 patients (26.6%), of which 34 females (23.9%) and 21 males (32.3%). An exclusively familial history of AD was found in nine patients (4.3%; 8 F, 5.6%; 1 M, 1.5%). Overall, an atopic background was present in 66.7% of our study population (138/207: 97 females, 68.3%, and 41 males, 63.1%) (Table 1).

Most patients (57.0%) were occupationally at risk (Table 2). The association between onset/worsening of CHE and occupation was observed in 84 patients (40.6%). On average, our patients had been doing their current work for more than 13 years, with large variability (164.4 ± 150.0 months, range 2–600). A previously different work was declared by 73 patients (32.3%) (wetwork in 24 cases, non‐wetwork but at risk of hand eczema in 11, non‐wetwork and not at risk of HE in 38), who reported doing it for a period ranging from less than 1 year to about 50 years (mean 43.1 ± 100.8 months).

**TABLE 2:** Current occupations reported by patients with chronic hand eczema.

Work categories	Total, *n*. (%)	Females, *n*. (%)	Males, *n*. (%)
*Wetworkers*	*94 (45.4)*	*76 (53.5)*	*18 (27.7)*
Housewives	29 (14.0)	29 (20.4)	0 (0.0)
Hairdressers, beauticians	21 (10.1)	16 (11.3)	5 (7.7)
Healthcare workers (nurses, nursing assistants, doctors, surgeons, other healthcare workers)	21 (10.1)	18 (12.7)	3 (4.6)
Food workers (cooks, bartenders, pastry chefs, food clerks, fishmongers, etc.)	15 (7.3)	11 (7.7)	4 (6.2)
Metalworkers who use cutting oils	6 (2.9)	0 (0.0)	6 (9.2)
Other wetworkers (e.g., fishermen, workers in animal farms, greenhouses, stables, pet stores, etc.)	2 (1.0)	2 (1.4)	0 (0.0)
*Non wetworkers at risk for hand eczema*	*24 (11.6)*	*3 (2.1)*	*21 (32.3)*
Construction workers (bricklayers, tilers, flooring contractors, plumbers, painters, etc.)	6 (2.9)	0 (0.0)	6 (9.2)
Chemical industry workers	3 (1.4)	1 (0.7)	2 (3.1)
Farmers and labourers	3 (1.4)	1 (0.7)	2 (3.1)
Metalworkers, welders, etc.	3 (1.4)	0 (0.0)	3 (4.6)
Carpenters	2 (1.0)	0 (0.0)	2 (3.1)
Electronic industry workers	1 (0.5)	0 (0.0)	1 (1.5)
Assemblers of rubber artefacts, printed circuit boards, etc.	1 (0.5)	0 (0.0)	1 (1.5)
Mechanics, coachbuilders, tire dealers, etc.	1 (0.5)	0 (0.0)	1 (1.5)
Other manual workers	4 (1.9)	1 (0.7)	3 (4.6)
*Other workers*	*89 (43.0)*	*63 (44.4)*	*26 (40.0)*
Office workers (of all kinds)	34 (16.4)	26 (18.3)	8 (12.3)
Students	19 (9.2)	15 (10.6)	4 (6.2)
Retirees	9 (4.3)	5 (3.5)	4 (6.2)
Merchants and vendors (not in wetwork categories)	5 (2.4)	4 (2.8)	1 (1.5)
Teachers	5 (2.4)	4 (2.8)	1 (1.5)
Healthcare professionals not devoted to wetwork	4 (1.9)	3 (2.1)	1 (1.5)
Computer technicians and other technicians	2 (1.0)	0 (0.0)	2 (3.1)
Managers (of all types)	1 (0.5)	0 (0.0)	1 (1.5)
Others (e.g., craftsmen not in contact with irritants, military personnel, entertainment workers, sports workers, etc.)	10 (4.8)	6 (4.2)	4 (6.2)

Distribution of the current work types significantly varied (*p* < 0.001) between males and females: wetwork was more frequent among females than males (53.5% vs. 27.7%, respectively), works classified as non‐wetwork but at risk for CHE were more frequent among males than females (32.3% vs. 2.1%, respectively), while works not at risk for CHE were almost equally distributed in females and males (44.4% and 40.0%, respectively). The 73 patients who reported a previously different work were 19 females and 5 males for wetwork, 2 females and 9 males for works not classified as wetwork but at risk for CHE, 30 females and 8 males for works not at risk for CHE (overall *p =* 0.0006). No significant difference concerning the age at enrollment visit or the age of onset of the disease was found between patients with and without a history of current or past wetwork. This was confirmed comparing the male and female subgroups.

Most patients reported doing housework activities (cooking, cleaning) every day (122, 58.9%) or sometimes (57, 27.5%). Among the former, females were largely more than males (107 females, 75.4% vs. 15 males, 23.1%, *p* < 0.001); in particular, 29/142 (20.4%) females were housewives. Among the others, 34 cumulated daily housework to other wetwork occupations and 2 to non‐wetwork occupations at risk of HE. Thirty‐six patients (17.4%), mainly females (27, 19.0%) took daily care of children of less than 3 years of age. No significant age difference was recorded between patients who took daily care of children and those who did not (42.8 ± 13.3 vs. 40.8 ± 16.0 years, *p =* 0.28). Frequent (11–20 times/day) or very frequent (> 20 times/day) handwashing was reported by 40.6% and 20.3% of patients, respectively, and more frequently by females (71.4% and 83.3%, respectively, *p =* 0.013, not significant after Benjamini‐Hochberg's correction).

Habitual practice of manual leisure activities that can expose hands to irritants and allergens, such as gardening and do‐it‐yourself activities, was reported by 81 patients (39.1%; 36 males and 45 females). No significant age difference was recorded between patients with a manual hobby and those who had not such activity (42.0 ± 16.9 vs. 40.6 ± 14.7 years, *p =* 0.58).

Other potential risk factors for HE reported in the literature and investigated in our study population were body mass index (BMI) and smoking (Table 1). Neither of them was significantly associated with CHE in our patients.

As per the ESCD guidelines [10] and per our study protocol, all patients underwent patch testing. A total of 99 patients (47.8%) had at least one positive patch test reaction (Table 3). In 50 of them (24.2% of the study population, 50.5% of sensitised subjects), patch test reactions were considered relevant for CHE. Exposure was occupational in 19 of these, non‐occupational in 24, mixed in 7. Patch test results, occupational exposure and allergens avoidability are also reported in Table 3. Methylisothiazolinone resulted the most frequent relevant sensitizer (12, 9 females and 3 males), followed by 2‐hydroxyethylmetacrylate (8, all females) and *p*‐phenylendiamine (8, 5 females and 3 males). In most cases (63/80, 78.8%) contact with the responsible allergen was considered avoidable. In four cases, contact sensitivity to a given allergen was already known by the patient because of a previous positive patch test, fully avoided in 1 (fragrance mix II), doubtfully avoided in 1 (nickel sulphate), not avoided in the other 2 (thiuram mix and *p*‐ter‐butyl‐phenol‐formaldehyde resin, respectively).

**TABLE 3:** Relevant and total positive patch test reactions, avoidable contact, and occupational exposure of relevant allergens.

Allergen	Relevant positive reactions (total positive reactions)				Avoidable contact of relevant allergen		Occupational contact of relevant allergen		
	+	++	+++	Total	Yes	No	Yes	No	In part
2‐Methyl‐4‐isothiazolin‐3‐one 0.2% aq.	2 (6)	6 (8)	4 (5)	12 (19)	11	1	4	6	2
p‐Phenylendiamine 1% pet.	0 (1)	5 (5)	3 (4)	8 (10)	5	3	5	3	0
2‐Hydroxyethylmetacrylate 1% pet.	0 (1)	7 (9)	1 (1)	8 (11)	5	3	3	5	0
Thiuram mix 1% pet.	0 (0)	6 (7)	1 (1)	7 (8)	6	1	5	2	0
Methylisothiazolinone/methylchloroisothiazolinone 0.02% aq.	2 (7)	3 (3)	2 (3)	7 (13)	6	1	1	4	2
Disperse dye mix 6.6% pet.	0 (2)	4 (4)	2 (4)	6 (10)	5	1	3	2	1
Nichel sulphate hexahydrate 5% pet.	2 (12)	2 (27)	2 (8)	6 (47)	3	3	2	1	3
Fragrance mix I 8% + sorbitan sesquioleate 5% pet.	2 (5)	1 (2)	0 (0)	3 (7)	3	0	1	1	1
Cobalt chloride hexahydrate 1% pet.	1 (2)	2 (6)	0 (0)	3 (8)	3	0	2	1	0
*Myroxylon pereirae* (Balsam of Peru) 25% pet.	2 (5)	1 (3)	0 (0)	3 (8)	3	0	0	2	1
Cocamidopropylbetaine 1% pet.	1 (2)	1 (1)	0 (0)	2 (3)	1	1	1	1	0
Fragrance mix II 14% pet.	0 (0)	1 (1)	1 (2)	2 (3)	2	0	1	1	0
N‐Isopropyl‐N′‐phenyl‐p‐phenylendiamine 0.1% pet.	0 (2)	0 (0)	2 (2)	2 (4)	0	2	2	0	0
Colophony 20% pet.	1 (2)	1 (3)	0 (2)	2 (7)	2	0	1	1	0
Benzisothiazolinone 0.1% aq.	0 (0)	1 (1)	0 (0)	1 (1)	0	1	1	0	0
Compositae mix II 6% pet.	0 (0)	1 (1)	0 (0)	1 (1)	1	0	1	0	0
Hydroxymethylpentylcyclohexenecarboxaldehyde (Lyral) 5% pet.	0 (0)	1 (1)	0 (0)	1 (1)	1	0	1	0	0
Lanolin alcohol 30% pet.	1 (1)	0 (0)	0 (0)	1 (1)	1	0	0	1	0
Parabens mix 16% pet.	0 (0)	1 (2)	0 (0)	1 (2)	1	0	0	1	0
Budesonide 0.01% pet.	1 (2)	0 (0)	0 (0)	1 (2)	1	0	0	1	0
Mercaptobenzothiazole mix 2% pet.	0 (0)	1 (3)	0 (0)	1 (3)	1	0	1	0	0
p‐ter‐Butyl‐phenol‐formaldehyde resin 1% pet.	1 (2)	0 (1)	0 (0)	1 (3)	1	0	1	0	0
Potassium bichromate 0.5% pet.	0 (2)	1 (2)	0 (0)	1 (4)	1	0	1	0	0
Formaldehyde 2% aq.	0 (1)	0 (2)	0 (0)	0 (3)	0	0	0	0	0
Sodium metabisulphite 1% pet.	0 (2)	0 (1)	0 (0)	0 (3)	0	0	0	0	0
2‐Mercaptobenzothiazole 2% pet.	0 (0)	0 (0)	0 (1)	0 (1)	0	0	0	0	0
3‐Dimethylamino‐1‐propylamine 1% aq.	0 (1)	0 (0)	0 (0)	0 (1)	0	0	0	0	0
Epoxy resin 1% pet.	0 (1)	0 (0)	0 (0)	0 (1)	0	0	0	0	0
Neomycin sulphate 20% pet.	0 (1)	0 (0)	0 (0)	0 (1)	0	0	0	0	0
Sorbitan sesquioleate 20% pet.	0 (1)	0 (0)	0 (0)	0 (1)	0	0	0	0	0
Caine mix 10% pet.	0 (0)	0 (0)	0 (0)	0 (0)	0	0	0	0	0
Tixocortol pivalate 1% pet.	0 (0)	0 (0)	0 (0)	0 (0)	0	0	0	0	0

Abbreviations: aq, water; pet, petrolatum.

Etiological subtypes are shown in Table 4. ACD and ICD subtypes were based on assessment of relevant allergic or irritant exposures. ICD was the most frequent (52.7%), followed by ACD (24.2%) and AD (16.9%). Twenty‐two patients had a mixed aetiology. In 36 patients (17.4%) the aetiology was unclear.

**TABLE 4:** Etiological and clinical subtypes of chronic hand eczema.

Subtypes	Total, *n*. (%)	Females, *n*. (%)	Males, *n*. (%)
*Etiologic subtypes*			
ICD	94 (45.4)	64 (45.1)	30 (46.2)
ACD	33 (15.9)	25 (17.6)	8 (12.3)
AD	22 (10.6)	14 (9.9)	8 (12.3)
ICD + ACD	9 (4.3)	6 (4.2)	3 (4.6)
ACD + AD	7 (3.3)	3 (2.1)	4 (6.2)
ICD + AD	5 (2.4)	5 (3.5)	0 (0.0)
ICD + ACD + AD	1 (0.4)	1 (0.7)	0 (0.0)
Unclear	36 (17.4)	24 (16.9)	12 (18.5)
*Clinical subtypes*			
Hyperkeratotic eczema	25 (12.1)	15 (10.6)	10 (15.4)
Acute recurrent vesicular eczema	20 (9.7)	16 (11.3)	4 (6.2)
Pulpitis	11 (5.3)	5 (3.5)	6 (9.2)
Nummular eczema	10 (4.8)	5 (3.5)	5 (7.7)
*Combined (etiological + clinical subtypes)*			
ICD + hyperkeratotic eczema	4 (1.9)	4 (2.8)	0 (0.0)
ACD + acute recurrent vesicular eczema	3 (1.4)	3 (2.1)	0 (0.0)
ACD + pulpitis	3 (1.4)	1 (0.7)	2 (3.1)
ACD + hyperkeratotic eczema	3 (1.4)	2 (1.4)	1 (1.5)
ICD + pulpitis	2 (1.0)	1 (0.7)	1 (1.5)
AD + acute recurrent vesicular eczema	2 (1.0)	1 (0.7)	1 (1.5)
ICD + nummular eczema	1 (0.5)	1 (0.7)	0 (0.0)
ICD + acute recurrent vesicular eczema	1 (0.5)	0 (0.0)	1 (1.5)
ICD + pulpitis + hyperkeratotic eczema	1 (0.5)	0 (0.0)	1 (1.5)
ICD + pulpitis + acute recurrent vesicular eczema	1 (0.5)	1 (0.7)	0 (0.0)
ACD + pulpitis + acute recurrent vesicular eczema	1 (0.5)	1 (0.7)	0 (0.0)
ACD + AD + nummular eczema	1 (0.5)	0 (0.0)	1 (1.5)
ACD + AD + acute recurrent vesicular eczema	1 (0.5)	1 (0.7)	0 (0.0)
ACD + ICD + hyperkeratotic eczema + acute recurrent vesicular eczema	1 (0.5)	0 (0.0)	1 (1.5)
AD + nummular eczema	1 (0.5)	0 (0.0)	1 (1.5)

Abbreviations: ACD, allergic contact dermatitis; AD, atopic dermatitis; ICD, irritant contact dermatitis.

Clinical subtypes, as defined by guidelines [10], were recognised only in 60/207 (29.0%) of patients. The most frequent were hyperkeratotic eczema (12.1%) and acute recurrent vesicular eczema (9.7%), followed by pulpitis (5.3%) and nummular eczema (4.8%). Most patients had varying degrees and combinations of the typical signs of CHE (erythema, scaling, vesicles, oedema, papules, hyperkeratosis, lichenification, and fissures). Scaling, erythema, and fissures were the most common clinical signs, observed in 93.7%, 88.9%, and 73.4% of patients, respectively.

A combined etiological‐morphological classification was given in 26 cases (12.6%). Comparison between the number of males and females for each etiological or clinical subtypes showed no significant differences.

**FIGURE 1:**
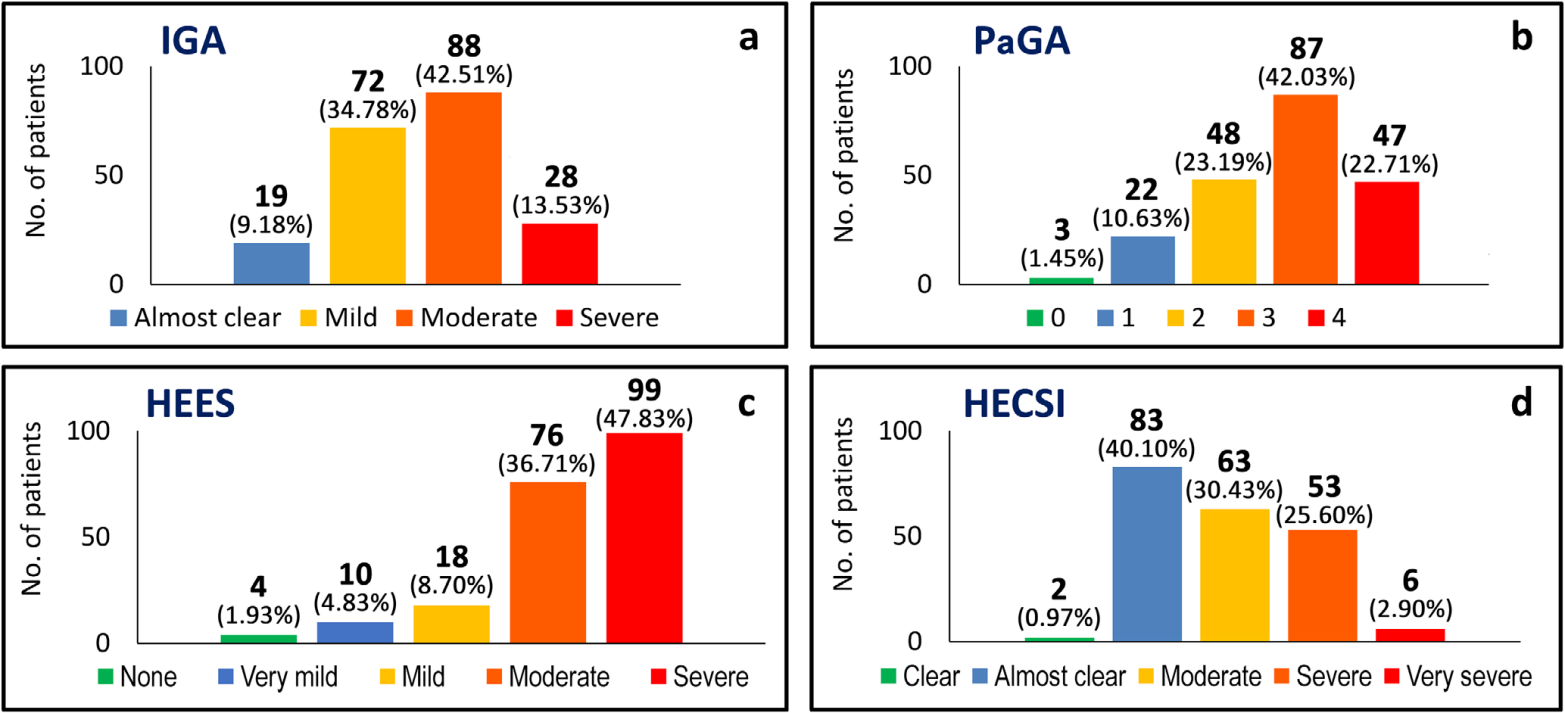
Objective and subjective assessment of chronic hand eczema severity in the study population. (a) IGA‐CHE (Investigator's Global assessment‐Chronic Hand Eczema), (b) PaGA (Patient's Global Assessment), (c) HEES (Hand Eczema Extent Score), (d) HECSI (Hand ECzema Severity Index).

Subjective and objective severity scores are reported in Figure 1. Most cases (116/207, 56.0%) were classified as moderate/severe according to the IGA‐CHE score (Figure 1a). PaGA score was slightly worse, with 134 cases classified with the two highest scores of the scale (Figure 1b). On a scale from 0 to 4, mean IGA‐CHE and PaGA were 2.6 ± 0.8 and 2.7 ± 1.0, respectively. Objective measurements showed that the extension of the disease (Figure 1c), measured by the HEES, was often large (197/207, 95.2%, classified as moderate or severe), while severity (Figure 1d), measured by HECSI, was moderate to very severe in 122/207 cases (58.9%). The mean HEES in our study population was 25.4 ± 14.5, while the mean HECSI was 30.3 ± 32.7.

In addition to hands, eczema also affected one or more other body areas in one third of cases (69/207): limbs in 30 (14.5%), trunk in 25 (12.1%), face in 16 (7.7%), plantar eczema in 14 (6.8%), and diffuse eczema in 3 (1.4%). Very severe CHE (HECSI > 38) was not significantly associated with involvement of other body areas in comparison to exclusive involvement of hands (33.9% vs. 33.1%, respectively, *p =* 0.91).

Concerning pruritus and pain, both measured using NRS, means were 6.1 ± 3.1 and 5.0 ± 3.2, respectively. All severity parameters were not significantly different between females and males.

Based on multivariate analysis (dependent variable HECSI > 38, independent variables: sex, atopy, wetwork and non‐wetwork occupations, relevant contact allergy, concomitant eczematous lesions in other body areas), severe CHE was significantly associated only with relevant positive patch test reactions (*p =* 0.045).

**FIGURE 2:**
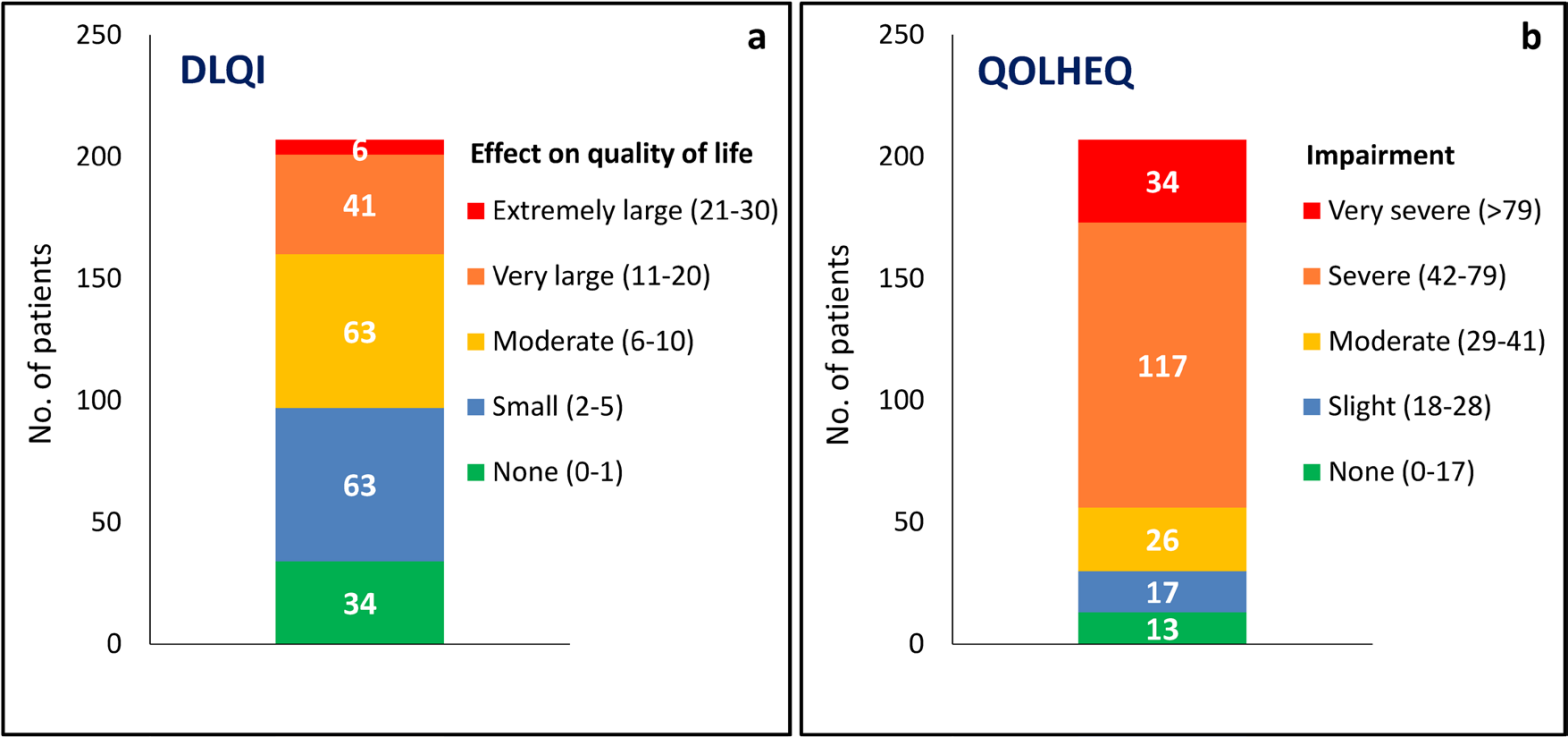
Quality of life measurements in the study population. (a) DLQI (Dermatology Life Quality Index), (b) QOLHEQ (Quality of Life in Hand Eczema Questionnaire).

The impact of CHE on QoL is graphically shown in Figure 2. DLQI scores were in the moderate/large/extremely large range in 110 cases (53.1%), and the overall mean of this parameter was 7.1 ± 5.8 (Figure 2a). The QOLHEQ scores were in the moderate/severe/very severe range in 177 patients (85.5%), with a particularly high frequency of severe cases (*n* = 117, 56.5%), and the overall mean score was 56.2 ± 24.9 (Figure 2b). Analysis of the four domains of QOLHEQ showed that ‘symptoms’ and ‘treatment‐prevention’ were the most affected ones, with a mean score of 16.8 ± 5.6 and 14.3 ± 5.8, respectively, on a scale from 0 to 28, while mean scores for ‘emotions’ and ‘functioning’ were 13.5 ± 8.1 and 11.7 ± 8.2, respectively, on a scale from 0 to 32. DLQI, total QOLHEQ, and QOLHEQ domain scores did not show significant differences between females and males.

Regarding the impact on working activity, this was documented in 36/207 (17.4%): in 19 (9.2%), in 10 (4.8%), and in 7 (3.4%) absence for less than 7 days, between 7 and 21 days, and for more than 21 days, respectively. Most patients (141/207, 68.1%) reported no absence from work because of CHE in the last year, while the question was not applicable in 30 cases (14.5%).

Full details about past and/or current treatments and the clinical response are shown in Table 5. The great majority of patients (93.7%) used emollients and barrier repair creams. Topical corticosteroids were used for past and/or current manifestations in almost all cases (201/207, 97.1%). However, they often proved inadequate: low/medium potency corticosteroids induced partial/temporary response, no response or adverse effects leading to drug withdrawal in 130/182 patients (71.4%) and high potency corticosteroids in 114/140 patients (81.4%). Only 3 patients (2 males and 1 post‐menopausal female) took/had taken alitretinoin.

**TABLE 5:** Reported clinical response to past and/or current therapies for chronic hand eczema.

Treatments		Results of treatment, *n*. (%)			
	Treated subjects *n*. (%)	Effective *n*. (%)	Partial/temporary *n*. (%)	None/Adverse^a^ *n*. (%)	Unknown *n*. (%)
*Topical treatments*					
Topical corticosteroids					
Low/medium potency	182 (87.9)	47 (22.7)	109 (52.7)	21 (10.1)	5 (2.4)
High potency	140 (67.6)	18 (8.7)	99 (47.8)	15 (7.2)	8 (3.9)
Tacrolimus	21 (10.1)	6 (2.9)	7 (3.4)	4 (1.9)	4 (1.9)
Pimecrolimus	13 (6.3)	1 (0.5)	6 (2.9)	2 (1.0)	4 (1.9)
*Systemic treatments*					
Oral corticosteroids	61 (29.5)	30 (14.5)	21 (10.1)	6 (2.9)	4 (1.9)
Cyclosporin	7 (3.4)	2 (1.0)	2 (1.0)	3 (1.4)	—
Other systemic immunosuppressants (methotrexate, azathioprine)	5 (2.4)	2 (1.0)	1 (0.5)	2 (1.0)	—
Alitretinoin	3 (1.4)	2 (1.0)	—	1 (0.5)	—
Biologics	1 (0.5)	1 (0.5)	—	3 (1.4)	—
Phototherapy	5 (2.4)	3 (1.4)	—	—	2 (1.0)

^a^ Includes cases of no clinical response and treatment withdrawal because of adverse effects.

The results of the short survey on the usefulness and ease of use of the CRF are shown in Table 6. The ICC was 0.8, suggesting a good inter‐rater agreement [33]. Expert dermatologists participating in the study generally scored the CRF as complete, intuitive, and easy to fill in (scores between 4 and 5) but requiring a time not always fully compatible with real‐setting clinical activity (between 10 and 20 min per patient). The most demanding tasks were to assess HEES and HECSI scores and the relevance of positive patch test reactions.

**TABLE 6:** Questionnaire results on usefulness and ease of use of the used case report form.

Questions	Score (mean ± SD)
Degree of agreement with each of the following sentences (from 1: total disagreement, to 5: complete agreement)	
The CRF allows for a comprehensive classification of the patient with CHE	4.6 ± 0.5
The CRF is intuitive and easy to fill in	4.2 ± 0.6
The time required to fill in the CRF is compatible with clinical activity	3.2 ± 0.8
Number of minutes to complete the CRF for a single patient	13.9 ± 3.8
Ease to fill in the following questionnaire sections (from 1: I do not agree, it is difficult, to 5: I totally agree, it is easy)	
Collection of data on exogenous and occupational risk factors	4.7 ± 0.5
HECSI	4.0 ± 0.7
HEES	3.9 ± 0.7
Quality of life assessment	4.6 ± 0.7
Definition of etiological diagnosis	4.3 ± 0.7
Definition of morphological diagnosis	4.2 ± 0.6
Assessing whether positive patch reactions tests were relevant for CHE	4.0 ± 0.7

Abbreviation: SD, standard deviation.

## 4 Discussion

This study aimed to create a simple and sufficiently accurate tool to systematically collect data of CHE patients, suitable for clinical practice and real‐life studies, in view of the imminent availability of new treatments that ideally require a targeted approach. This is not an easy task, because classifying CHE is complex due to its wide variety of etiologies and clinical manifestations, often overlapping [1, 3, 12, 34].

As highlighted by recent expert reviews, until reliable biomarkers become available for clinical practice, a comprehensive diagnostic workup is needed that should include detailed medical history, clinical examination, meticulous assessment of possible environmental triggers and patch testing to identify relevant contact allergens [1, 3, 17, 35, 36]. The CRF used in this study was designed to accurately register the key aspects of this kind of workup and included six validated scoring systems to quantify signs, symptoms, and disease severity plus two quality of life questionnaires to evaluate the burden of disease. The participating centres generally scored it as complete and easy to fill in, although requiring a time not always fully compatible with clinical activity.

As for the results of the study, with respect to demographics and constitutional/environmental risk factors, our patients' data were substantially in agreement with those reported in other European studies investigating chronic hand eczema, despite substantial heterogeneity in study design, population selection, and data collection methods [1, 3, 37, 38, 39, 40, 41, 42]. Large population‐based surveys conducted in Northern and Western Europe have provided robust estimates of the prevalence and overall burden of CHE in the general population (Table 7). Differences may reflect different patients' populations being studied. For example, the mean age of CHE patients varies across studies: it is often in the late 30s to early 50s, but some large registries as well as a recent SIDAPA study report mean ages around 47–48 years [37, 38, 39, 40, 41, 42, 43, 44].

**TABLE 7:** Population based surveys conducted in Northern and Western Europe on chronic hand eczema.

Author, year	Country	Study design	Population	N. patients	Data collection	Standardised CRF/unified clinical record	Main outcomes
Apfelbacher et al., 2011 [42]	Germany	Prospective clinical registry	CHE patients in specialised centres	515	Clinician + patient reported outcomes	Yes	Clinical and etiological characterisation
Cazzaniga et al., 2018 [41]	Switzerland	Prospective clinical registry	CHE patients in specialised centres	199	Clinician + patient reported outcomes	Yes	Prognostic factors, quality of life
Apfelbacher et al., 2019 [40]	Germany	Prospective longitudinal registry	CHE patients in specialised centres	1281	Clinician + patient reported outcomes	Yes	Disease course, long‐term outcomes
Voorberg et al., 2022 [39]	The Netherlands	Population‐based, cross‐sectional	General adult population	57 796	Patient‐reported survey	No	Prevalence, severity (self‐reported)
Thein et al., 2025 [37]	Denmark	Population‐based, cross‐sectional	General adult population	11 166	Patient‐reported survey	No	Prevalence, disease characteristics
Apfelbacher et al., 2025 [38]	Canada, France, Germany, Italy, Spain, UK	Multinational, population‐based, cross‐sectional	General adult population	60 131	Patient‐reported online survey	No	Multinational prevalence estimates
Our study	Italy	Multicentre, observational, cross‐sectional	CHE patients in specialised centres	207	Clinician + patient reported outcomes	Yes	Clinical and etiological characterisation, severity, burden of disease

Abbreviation: CHE, chronic hand eczema.

The mean age of our study population was 41.1 ± 15.5 years. The age of CHE onset varied widely but on average it was 35.7 ± 16.6 years. Only in 11 atopic patients HE onset dated from childhood, a recognised risk factor for CHE. The mean disease duration was 6.0 ± 8.1 years. In accordance with the literature, the majority (> 2/3) of our patients were females and this appeared to be related to higher environmental exposures to irritants, including more frequent handwashing and time spent on occupational/domestic wetwork and care of small children [1, 3, 42]. Noteworthy, also lower age of CHE onset and lower educational level, two other factors independently associated with CHE, were more frequent in women than in men.

Overall, 57.0% of our patients worked in occupations, both wetwork and non‐wetwork, considered at risk for CHE due to frequent contact with irritants and/or allergens, in line with the literature [1, 42, 45]. Habitual practice of manual leisure activities that also can expose hands to irritants and allergens was reported by 39.1% of our patients.

Contact allergy, a recognised risk factor associated with HE, was identified by positive patch tests in 47.8% of patients. However, only 24.2% of patients had relevant positive reactions for CHE and were classified etiologically as having ACD. This percentage is considerably lower than that reported in a previous SIDAPA study (48.1%) [44], where consecutive patients specifically referred for patch testing were analysed. In the present study, methylisothiazolinone, 2‐hydroxyethylmetacrylate, and *p*‐phenylenediamine were the most frequent relevant positive patch test allergens. Of note, based on multivariate analysis, relevant positive patch test reactions were significantly associated with severest CHE, in line with previous studies [46, 47]. In 4/50 patients with relevant positive patch test reactions, sensitization to the relevant allergen(s) was already known by the patients, but contact with the allergen(s) had not been avoided. It is important to identify this subset of patients: on one side non avoidance can explain the persistence/aggravation of CHE despite a correct diagnosis of ACD, on the other side, treatment with newer drugs may be particularly indicated where strict avoidance strategies are not possible, similarly to what has been described, for example, in severe cases of airborne contact dermatitis [48, 49].

As for AD, the strongest established constitutional risk factor for HE [3, 37], current or past AD was found in 27.1% and 10.1% of our patients, respectively. In 57.1% of the former, other body sites beside hands were involved. This is in accordance with the observation by other authors that many patients with active AD develop eczema on their hands, and that HE can be the only or predominant manifestation of AD or part of a more diffuse disease [14, 42, 50]. Interestingly, an atopic background was present in two thirds of our study population, suggesting, in line with other studies [8], that all atopic diseases, not only AD, may be associated with CHE.

Based on evaluation of the above factors, the disease was etiologically classified as ICD in 52.7% of patients, ACD in 24.2% and AD in 16.9%. Twenty‐two of these 184 patients (12.0%) had a mixed aetiology. Overall, a final etiological diagnosis was formulated in 82.6% of patients while in 17.4% the aetiology was doubtful/undefined.

From a clinical point of view, only 29.0% of patients could be morphologically classified as a specific clinical subtype, the most frequent ones being hyperkeratotic eczema and acute recurrent vesicular eczema, with no significant relationship with specific etiological subtypes. The remaining patients showed the typical signs of HE, the most common ones being scaling, erythema, and fissures, suggesting that there is a need to revise the guidelines on CHE, especially regarding the classification of clinical subtypes.

In most cases the disease was moderate to severe according to HECSI, IGA‐CHE, and PaGA scores, with rates that varied from 58.9% based on HECSI to 56.0% based on IGA‐CHE, while the patient's self‐evaluation by the PaGA resulted in 64.7% of cases rated with the two highest scores of the scale. Extension of the disease was often large. Based on HEES scores, it was classified as moderate to severe in 95.2% of patients. In addition to hands, eczematous lesions also affected one or more other body areas in one third of cases (69 patients), while 18 had ACD with possible autoeczematisation or concomitant exposure of other body sites to allergens. According to the literature, patients with involvement of other body areas tend to have more severe CHE [3, 51]. This was not true in our study population, where severe/very severe CHE cases with or without concomitant body involvement were equally represented (33.9% vs. 33.1%, *p =* 0.91).

In accordance with the literature [3, 41, 52], the impact of CHE on the quality of life of our patients was considerable. Based on QOLHEQ scoring, it ranked from moderate to very severe in as many as 85.5% of cases, with a particularly high frequency of severe impairment (56.5%). ‘Symptoms’ and ‘treatment‐prevention’ were the most affected domains. Absenteeism because of CHE was reported by 17.4% of patients.

Regarding treatments, overall sustained past and/or present efficacy of at least one pharmacological treatment was reported by 45.4% of our patients, while in more than half of the study population effective and safe therapy was not available. Of note, in our patients, the mean NRS value of pruritus, a parameter considered of major importance to measure the outcomes of CHE treatments [41, 53], was quite high (6.1 ± 3.1). As for systemic therapy, aside from the ‘as needed use’ of systemic corticosteroids in 29.6% of patients, treatment with other systemic drugs and/or phototherapy was reported only in 10.1% of the total study population and in 19.6% of patients refractory to potent topical corticosteroids. These percentages are low compared to data reported in the literature (around 36.5% of severe cases) [54]. Surprisingly, only 3 patients took/had taken alitretinoin, the only systemic agent currently approved in Italy for the treatment of severe CHE. The possible reasons are reluctance of female patients in childbearing age (37.3% of our patients with severe CHE) to take this drug, and bureaucratic hurdles hampering its prescription.

Our data confirm the large unmet need for effective and safe treatments in patients with moderate to severe CHE. Newer drugs that are becoming available for these patients, like the topical pan‐JAK inhibitor delgocitinib, are raising high expectations. However, we should learn to use them in a tailored way, and this represents a significant challenge. In the case of delgocitinib, as it targets several cytokine pathways, it may ideally benefit different etiological and clinical CHE subtypes but data on its effectiveness on the different subtypes are lacking. This is an important issue that needs careful assessment in future real‐life studies [55].

The CRF designed for the present study allowed an accurate diagnostic workup leading to etiological CHE classification in 82.6% (171/207) of patients. In expert hands, it can become a useful tool that may facilitate tailored treatment strategies and optimal evaluation of their results. At present, this kind of approach, although not ideal, seems a reliable way to try and compare the effect of different treatments on patients with different CHE subtypes in real‐life settings.

Our study has some limitations. Firstly, in the case of ACD, some relevant contact allergies may have gone undiagnosed because patch tests with integrative series or ROAT with patients' own products were not homogeneously performed. Furthermore, the relevance assessment of positive patch tests, based on clinical history, exposure source recognition, and clinical features, was sometimes doubtful and could not be confirmed by an adequate clinical‐etiological follow‐up. Secondly, as previously stated [34], the diagnosis of ICD is subject to misclassification due to the lack of diagnostic tests. In this study, the diagnosis of ICD was based on a set of detailed questions evaluating activities (occupational/domestic/leisure) that expose the hands to irritants. However, specific questions about some specific factors, in particular gloves, were not included for the sake of brevity. They might be added to the CRF for future studies. Furthermore, our study analyses a relatively small population of patients in a relatively short period of time, and the results need to be further verified with larger prospective studies. Finally, when it was initially designed, the main purpose of the CRF was broad patient classification and basic data collection, but its application for the follow‐up of treatments with new drugs needs updates for more detailed treatment response monitoring.

In conclusion, the long‐term use of newer drugs is likely to entail significant costs and its possible side effects are not yet known [56]. However exciting, the advent of these drugs does not exempt us from doing every possible effort to look for relevant contact irritants/allergens and to educate patients to avoid them through appropriate prevention measures.

## Author Contributions

**Rosella Gallo:** conceptualization, data curation, formal analysis, methodology, validation, visualisation, writing – original draft, writing – review and editing. **Fabrizio Guarneri:** conceptualization, data curation, formal analysis, methodology, software, investigation, validation, visualisation, writing – original draft, writing – review and editing. **Katharina Hansel:** conceptualization, methodology, investigation, validation, visualisation, writing – original draft, writing – review and editing. **Luca Stingeni:** conceptualization, methodology, investigation, validation, visualisation, writing – original draft, writing – review and editing. **Maddalena Napolitano:** conceptualization, methodology, investigation, validation, visualisation, writing – review and editing. **Cataldo Patruno:** conceptualization, methodology, investigation, validation, visualisation, writing – review and editing. **Monica Corazza:** conceptualization, methodology, investigation, validation, visualisation, writing – review and editing. **Alessandro Borghi:** investigation, validation, visualisation, writing – review and editing. **Ilaria Trave:** investigation, validation, visualisation, writing – review and editing. **Emanuela Martina:** conceptualization, methodology, investigation, validation, visualisation, writing – review and editing. **Maria Michela Lauriola:** investigation, validation, visualisation, writing – review and editing. **Rosanna Rita Satta:** investigation, validation, visualisation, writing – review and editing. **Caterina Foti:** investigation, validation, visualisation, writing – review and editing. **Paolo Romita:** conceptualization, methodology, investigation, validation, visualisation, writing – review and editing. **Roberta Giuffrida:** investigation, validation, visualisation, writing – review and editing. **Silvia Mariel Ferrucci:** conceptualization, data curation, formal analysis, methodology, investigation, validation, visualisation, writing – original draft, writing – review and editing.

## Conflicts of Interest

R.G. has no conflicts of interest to declare; F.G. has served as a member of an advisory board on hand eczema for Leopharma; K.H. has served as a member of advisory board or speaker for Abbvie, Almirall, Amgen, BMS LeoPharma, Pfizer, Eli Lilly, Sanofi Regeneron; L.S. has been principal investigator in clinical trials sponsored by and/or a member of advisory board or speaker for AbbVie, Almirall, Bristol Meyer Squibb, Celgene, Eli Lilly, Janssen, Leo Pharma, Novartis and Sanofi Genzyme; M.N. has no conflicts of interest to declare; C.P. acted as consultant, speaker, advisory board member, and/or investigator for Abbvie, Almirall, Amgen, Galderma, La Roche‐Posay, LeoPharma, Lilly, Novartis, Pfizer, Pierre Fabre, Regeneron, Sanofi; M.C. has served as principal investigator in clinical trials sponsored by and/or a member of advisory board or speaker for Almirall, Novartis, AbbVie, Eli Lilly, Sanofi Regeneron, Pfizer, Janssen, Leo Pharma; A.B. has served as principal investigator in clinical trials sponsored by and/or a member of advisory board or speaker for Almirall, Bristol Meyer Squibb, AbbVie, Eli Lilly, Novartis, Sanofi Regeneron, Janssen, Leo Pharma and Incyte; I.T. acted as consultant for Abbvie, Leopharma, Almirall, Novartis, Galderma, and as investigator in studies sponsored by Abbvie and Sanofi; E.M. has served as a member of advisory board, speaker or investigator for Abbvie, Pfizer, Novartis, Sanofi Regeneron; M.M.L. has no conflicts of interest to declare; R.R.S. has no conflicts of interest to declare; C.F. received honorarium for partecipation at advisory boards and presentation from Abbvie, Amgen, Almirall, Ely Lilly, Pfizer, Sanofi, Leo Pharma, Incyte, Novartis; P.R. acted as speaker/advisory board for Sanofi, Abbvie, Almirall, Pfizer, Leopharma, UCB Pharma, Lilly, Amgen, Novartis; S.M.F. has served as a member of advisory board, speaker or principal investigator for Abbvie, Almirall, Amgen, Bayer, Leo Pharma, Incyte, Galderma, Pfizer, Eli Lilly, Sanofi Regeneron.

## Supporting information


**Table S1:** Case Report Form (CRF) specifically designed for CHE patients.
**Table S2:** Questionnaire to evaluate the usefulness and ease of use of the Case Report Form (CRF).

## Data Availability

The data that support the findings of this study are available from the corresponding author upon reasonable request.

## References

[1] S. Weidinger and N. Novak , “Hand Eczema,” Lancet 404, no. 10470 (2024): 2476–2486. 39615508 10.1016/S0140-6736(24)01810-5

[2] E. Weisshaar , “Chronic Hand Eczema,” American Journal of Clinical Dermatology 25, no. 6 (2024): 909–926. 39300011 10.1007/s40257-024-00890-z PMC11511713

[3] S. Molin , E. Guttman‐Yassky , J. P. Thyssen , and A. Bewley , “Chronic Hand Eczema, Real World, and Patient Centricity: A Narrative Review,” Acta Dermato‐Venereologica 105 (2025): adv42596. 40171832 10.2340/actadv.v105.42596 PMC11977413

[4] A. S. Quaade , A. B. Simonsen , A. S. Halling , J. P. Thyssen , and J. D. Johansen , “Prevalence, Incidence, and Severity of Hand Eczema in the General Population – A Systematic Review and Meta‐Analysis,” Contact Dermatitis 84, no. 6 (2021): 361–374. 33548072 10.1111/cod.13804

[5] A. S. Quaade , F. Alinaghi , J. B. Dietz , C. Y. Erichsen , and J. D. Johansen , “Chronic Hand Eczema: A Prevalent Disease in the General Population Associated With Reduced Quality of Life and Poor Overall Health Measures,” Contact Dermatitis 89, no. 6 (2023): 453–463. 37634937 10.1111/cod.14407

[6] P. A. Cortesi , L. Scalone , A. Belisari , et al., “Cost and Quality of Life in Patients With Severe Chronic Hand Eczema Refractory to Standard Therapy With Topical Potent Corticosteroids,” Contact Dermatitis 70, no. 3 (2014): 158–168. 24102212 10.1111/cod.12130

[7] S. M. D. Ruff , K. A. Engebretsen , C. Zachariae , et al., “The Association Between Atopic Dermatitis and Hand Eczema: A Systematic Review and Meta‐Analysis,” British Journal of Dermatology 178, no. 4 (2018): 879–888. 29172235 10.1111/bjd.16147

[8] M. Koskelo , S. P. Sinikumpu , J. Jokelainen , and L. Huilaja , “Risk Factors of Hand Eczema: A Population‐Based Study Among 900 Subjects,” Contact Dermatitis 87, no. 6 (2022): 485–491. 35980390 10.1111/cod.14205 PMC9805011

[9] W. Jamil , Å. Svensson , A. Josefson , M. Lindberg , and L. Von Kobyletzki , “Incidence Rate of Hand Eczema in Different Occupations: A Systematic Review and Meta‐Analysis,” Acta Dermato‐Venereologica 102 (2022): adv00681. 35098319 10.2340/actadv.v102.360 PMC9631253

[10] J. P. Thyssen , M. L. A. Schuttelaar , J. H. Alfonso , et al., “Guidelines for Diagnosis, Prevention, and Treatment of Hand Eczema,” Contact Dermatitis 86, no. 5 (2022): 357–378. 34971008 10.1111/cod.14035

[11] D. Pesqué , J. F. Silvestre‐Salvador , A. C. Figueiredo , R. M. Pujol , M. Gonçalo , and A. M. Giménez‐Arnau , “A Review of Hand Eczema Subtypes: Clinical Features, Biomarkers and Treatment Strategies,” Contact Dermatitis 92, no. 6 (2025): 421–435. 39994885 10.1111/cod.14775

[12] A. S. Quaade , X. Wang , J. B. K. Sølberg , et al., “Inflammatory Plasma Signature of Chronic Hand Eczema: Associations With Aetiological and Clinical Subtypes,” Journal of the European Academy of Dermatology and Venereology 38, no. 6 (2024): 1101–1111. 38151335 10.1111/jdv.19742

[13] C. Dubin , E. Del Duca , and E. Guttman‐Yassky , “Drugs for the Treatment of Chronic Hand Eczema: Successes and Key Challenges,” Therapeutics and Clinical Risk Management 16 (2020): 1319–1332. 33408476 10.2147/TCRM.S292504 PMC7780849

[14] R. Bissonnette , R. B. Warren , A. Pinter , et al., “Efficacy and Safety of Delgocitinib Cream in Adults With Moderate to Severe Chronic Hand Eczema (DELTA 1 and DELTA 2): Results From Multicentre, Randomised, Controlled, Double‐Blind, Phase 3 Trials,” Lancet 404, no. 10451 (2024): 461–473. 39033766 10.1016/S0140-6736(24)01027-4

[15] M. Gooderham , S. Molin , R. Bissonnette , et al., “Long‐Term Safety and Efficacy of Delgocitinib Cream for up to 52 Weeks in Adults With Chronic Hand Eczema: Results of the Phase 3 Open‐Label Extension DELTA 3 Trial Following the DELTA 1 and 2 Trials,” Journal of the American Academy of Dermatology 93, no. 1 (2025): 95–103. 40081663 10.1016/j.jaad.2025.03.008

[16] G. R. Lee , M. Maarouf , A. K. Hendricks , D. E. Lee , and V. Y. Shi , “Current and Emerging Therapies for Hand Eczema,” Dermatologic Therapy 32, no. 3 (2019): e12840. 30693618 10.1111/dth.12840

[17] G. Ghezzi , C. Falcidia , G. Paolino , et al., “Chronic Hand Eczema (CHE): A Narrative Review,” Dermatology and Therapy (Heidelberg) 15, no. 4 (2025): 771–795. 10.1007/s13555-025-01365-7 PMC11971080 40064754

[18] H. R. Riva , T. Yoon , A. J. Hendricks , et al., “Dupilumab for Chronic Hand Eczema: A Systematic Review and Meta‐Analysis,” Dermatitis 37, no. 1 (2026): 20–41, 10.1089/derm.2024.0186. 39501849

[19] N. Asamoah , M. K. Branyiczky , A. Almuqrin , et al., “Efficacy and Safety of Dupilumab in Chronic Hand Eczema: A Systematic Review,” Archives of Dermatological Research 317, no. 1 (2025): 441. 39976781 10.1007/s00403-025-03947-z

[20] K. Sardana , S. Sharath , A. Khurana , et al., “Th1 and Th2 Cytokine Expression in Hyperkeratotic Chronic Hand Eczema and the Role of Tofacitinib a Oral JAK Inhibitor,” Archives of Dermatological Research 316, no. 10 (2024): 682. 39400740 10.1007/s00403-024-03438-7

[21] P. A. Jimenez , H. L. Sofen , R. Bissonnette , et al., “Oral Spleen Tyrosine Kinase/Janus Kinase Inhibitor Gusacitinib for the Treatment of Chronic Hand Eczema: Results of a Randomized Phase 2 Study,” Journal of the American Academy of Dermatology 89, no. 2 (2023): 235–242. 37094653 10.1016/j.jaad.2023.04.027

[22] A. Carlsson , Å. Svensson , C. D. Anderson , et al., “Scoring of Hand Eczema: Good Reliability of Hand Eczema Extent Score (HEES),” Acta Dermato‐Venereologica 97, no. 2 (2017): 193–197. 27563701 10.2340/00015555-2521

[23] E. Held , R. Skoet , J. D. Johansen , and T. Agner , “The Hand Eczema Severity Index (HECSI): A Scoring System for Clinical Assessment of Hand Eczema. A Study of Inter‐ and Intraobserver Reliability,” British Journal of Dermatology 152, no. 2 (2005): 302–307. 15727643 10.1111/j.1365-2133.2004.06305.x

[24] J. I. Silverberg , T. Agner , K. Baranowski , et al., “Validation of the Investigator Global Assessment of Chronic Hand Eczema (IGA‐CHE): A New Clinician Reported Outcome Measure of CHE Severity,” Archives of Dermatological Research 316, no. 4 (2024): 110. 38507100 10.1007/s00403-024-02818-3 PMC10955004

[25] M. Hald , N. K. Veien , G. Laurberg , and J. D. Johansen , “Severity of Hand Eczema Assessed by Patients and Dermatologist Using a Photographic Guide,” British Journal of Dermatology 156, no. 1 (2007): 77–80. 17199570 10.1111/j.1365-2133.2006.07565.x

[26] N. Q. Phan , C. Blome , F. Fritz , et al., “Assessment of Pruritus Intensity: Prospective Study on Validity and Reliability of the Visual Analogue Scale, Numerical Rating Scale and Verbal Rating Scale in 471 Patients With Chronic Pruritus,” Acta Dermato‐Venereologica 92, no. 5 (2012): 502–507. 22170091 10.2340/00015555-1246

[27] M. Worm , J. P. Thyssen , S. Schliemann , et al., “The Pan‐JAK Inhibitor Delgocitinib in a Cream Formulation Demonstrates Dose Response in Chronic Hand Eczema in a 16‐Week Randomized Phase IIb Trial,” British Journal of Dermatology 187, no. 1 (2022): 42–51. 35084738 10.1111/bjd.21146 PMC9350381

[28] L. Stingeni , L. Bianchi , E. S. Caroppo , et al., “The New Italian SIDAPA Baseline Series for Patch Testing (2023): An Update According to the New Regulatory Pathway for Contact Allergens,” Italian Journal of Dermatology and Venereology 159, no. 2 (2024): 83–104. 38650492 10.23736/S2784-8671.24.07733-8

[29] L. Stingeni , L. Bianchi , K. Hansel , et al., “Italian Guidelines in Patch Testing ‐ Adapted From the European Society of Contact Dermatitis (ESCD),” Giornale Italiano di Dermatologia e Venereologia 154, no. 3 (2019): 227–253. 30717577 10.23736/S0392-0488.19.06301-6

[30] A. Y. Finlay and G. K. Khan , “Dermatology Life Quality Index (DLQI)–A Simple Practical Measure for Routine Clinical Use,” Clinical and Experimental Dermatology 19, no. 3 (1994): 210–216. 8033378 10.1111/j.1365-2230.1994.tb01167.x

[31] R. F. Ofenloch , E. Weisshaar , A. K. Dumke , S. Molin , T. L. Diepgen , and C. Apfelbacher , “The Quality of Life in Hand Eczema Questionnaire (QOLHEQ): Validation of the German Version of a New Disease‐Specific Measure of Quality of Life for Patients With Hand Eczema,” British Journal of Dermatology 171, no. 2 (2014): 304–312. 24397866 10.1111/bjd.12819

[32] R. Gallo , L. Stingeni , M. Corazza , et al., “Translation and Pre‐Validation of the Quality of Life in Hand Eczema Questionnaire (QOLHEQ),” Italian Journal of Dermatology and Venereology 159, no. 6 (2024): 682–684. 39611422 10.23736/S2784-8671.24.08026-5

[33] T. K. Koo and M. Y. Li , “A Guideline of Selecting and Reporting Intraclass Correlation Coefficients for Reliability Research,” Journal of Chiropractic Medicine 15, no. 2 (2016): 155–163. 27330520 10.1016/j.jcm.2016.02.012 PMC4913118

[34] J. D. Johansen , M. Hald , B. L. Andersen , et al., “Classification of Hand Eczema: Clinical and Aetiological Types. Based on the Guideline of the Danish Contact Dermatitis Group,” Contact Dermatitis 65, no. 1 (2011): 13–21. 21658054 10.1111/j.1600-0536.2011.01911.x

[35] L. Scalone , P. A. Cortesi , L. G. Mantovani , et al., “Clinical Epidemiology of Hand Eczema in Patients Accessing Dermatological Reference Centres: Results From Italy,” British Journal of Dermatology 172, no. 1 (2015): 187–195. 24974982 10.1111/bjd.13220

[36] L. Stingeni , M. C. Fargnoli , F. Guarneri , et al., “Italian Expert Opinion on Chronic Hand Eczema: From Guidelines to Clinical Practice,” Dermatologic Therapy 15, no. 1 (2025): 75–93. 10.1007/s13555-024-01312-y PMC11785867 39607665

[37] D. Thein , J. T. Maul , S. Ribero , J. I. Silverberg , A. Egeberg , and J. P. Thyssen , “Prevalence and Characteristics of Chronic Hand Eczema Among Adults in Denmark: A General Population‐Based Study,” Contact Dermatitis 92, no. 5 (2025): 358–366. 39756812 10.1111/cod.14732

[38] C. Apfelbacher , A. Bewley , S. Molin , et al., “Prevalence of Chronic Hand Eczema in Adults: A Cross‐Sectional Survey of Over 60 000 Respondents From the General Population of Canada, France, Germany, Italy, Spain and the UK,” British Journal of Dermatology 192, no. 6 (2025): 1047–1054. 39797908 10.1093/bjd/ljaf020

[39] A. N. Voorberg , L. Loman , and M. L. A. Schuttelaar , “Prevalence and Severity of Hand Eczema in the Dutch General Population: A Cross‐Sectional, Questionnaire Study Within the Lifelines Cohort Study,” Acta Dermato‐Venereologica 102 (2022): adv00626. 34664079 10.2340/actadv.v101.432 PMC9631254

[40] C. J. Apfelbacher , R. F. Ofenloch , E. Weisshaar , et al., “Chronic Hand Eczema in Germany: 5‐Year Follow‐Up Data From the CARPE Registry,” Contact Dermatitis 80, no. 1 (2019): 45–53. 30246346 10.1111/cod.13113

[41] S. Cazzaniga , B. K. Ballmer‐Weber , N. Gräni , et al., “Chronic Hand Eczema: A Prospective Analysis of the Swiss CARPE Registry Focusing on Factors Associated With Clinical and Quality of Life Improvement,” Contact Dermatitis 79, no. 3 (2018): 136–148. 29943397 10.1111/cod.13041

[42] C. J. Apfelbacher , W. Akst , S. Molin , et al., “CARPE: A Registry Project of the German Dermatological Society (DDG) for the Characterization and Care of Chronic Hand Eczema,” Journal der Deutschen Dermatologischen Gesellschaft 9, no. 9 (2011): 682–688. 21564539 10.1111/j.1610-0387.2011.07694.x

[43] P. Bentz , C. Apfelbacher , W. Akst , et al., “Self‐Reported Versus Physician‐Reported Severity of Chronic Hand Eczema: Concordance Analysis Based on Data From the German Chronic Hand Eczema Patient Long‐Term Management Registry,” Acta Dermato‐Venereologica 103 (2023): adv00884. 36892509 10.2340/actadv.v103.5383 PMC10015412

[44] L. Stingeni , M. Napolitano , K. Hansel , et al., “Hand Eczema in Italian Patients Referred for Patch Testing: A Retrospective SIDAPA Study (2016–2023),” Contact Dermatitis 92, no. 1 (2025): 9–20. 39187476 10.1111/cod.14684

[45] S. Cazzaniga , C. Apfelbacher , T. Diepgen , et al., “Patterns of Chronic Hand Eczema: A Semantic Map Analysis of the CARPE Registry Data,” British Journal of Dermatology 178, no. 1 (2018): 229–237. 28498524 10.1111/bjd.15660

[46] M. Hald , T. Agner , J. Blands , et al., “Clinical Severity and Prognosis of Hand Eczema,” British Journal of Dermatology 160, no. 6 (2009): 1229–1236. 19416249 10.1111/j.1365-2133.2009.09139.x

[47] T. Agner , K. E. Andersen , F. M. Brandao , et al., “Contact Sensitisation in Hand Eczema Patients‐Relation to Subdiagnosis, Severity and Quality of Life: A Multi‐Centre Study,” Contact Dermatitis 61, no. 5 (2009): 291–296. 19878245 10.1111/j.1600-0536.2009.01630.x

[48] I. Rivera‐Ruiz , A. Gil‐Villalba , and F. J. Navarro‐Triviño , “Exploring New Horizons in Allergic Contact Dermatitis Treatment: The Role of Emerging Therapies,” Actas Dermo‐Sifiliográficas 116, no. 7 (2025): T731–T739. 40446917 10.1016/j.ad.2025.05.016

[49] R. Gallo , I. Trave , R. Castelli , G. Gasparini , and A. Parodi , “Follow‐Up of Patch Test Reactivity to Sesquiterpene Lactone Mix in a Patient Successfully Treated With Dupilumab for Severe Airborne Allergic Contact Dermatitis,” Contact Dermatitis 89, no. 2 (2023): 140–141. 37308168 10.1111/cod.14367

[50] J. I. Silverberg , B. Simpson , K. Abuabara , et al., “Prevalence and Burden of Atopic Dermatitis Involving the Head, Neck, Face, and Hand: A Cross Sectional Study From the TARGET‐DERM AD Cohort,” Journal of the American Academy of Dermatology 89, no. 3 (2023): 519–528. 37150299 10.1016/j.jaad.2023.04.052

[51] A. H. Petersen , J. D. Johansen , and M. Hald , “Hand Eczema – Prognosis and Consequences: A 7‐Year Follow‐Up Study,” British Journal of Dermatology 171, no. 6 (2014): 1428–1433. 25156938 10.1111/bjd.13371

[52] A. Bewley , S. Kalia , E. Jonsenet , et al., “Burden of Disease in Patients With Chronic Hand Eczema: Systematic Literature Reviews of Healthcare Resource Use and Health‐Related Quality of Life,” Value in Health 27, no. 12 (2024): S536.

[53] H. Rönsch , F. Schiffers , R. Ofenloch , et al., “Which Outcomes Should Be Measured in Hand Eczema Trials? Results From Patient Interviews and an Expert Survey,” Journal of the European Academy of Dermatology and Venereology 37, no. 6 (2023): 1199–1206. 36695080 10.1111/jdv.18923

[54] J. S. S. Ho and S. Molin , “A Review of Existing and New Treatments for the Management of Hand Eczema,” Journal of Cutaneous Medicine and Surgery 27, no. 5 (2023): 493–503. 37496489 10.1177/12034754231188325 PMC10617006

[55] M. L. A. Schuttelaar , “A New Avenue for Treatment of Chronic Hand Eczema,” British Journal of Dermatology 187, no. 1 (2022): 7–8. 10.1111/bjd.21604 PMC10286637 35490378

[56] S. Yang , N. Babaei , M. Cervantes , M. Gill , and J. J. Wu , “Reflections on Chronic Hand Eczema,” Lancet 405, no. 10486 (2025): 1228. 10.1016/S0140-6736(25)00341-1 40221161

