# Supplementary material for: Real‐Life Workup of Chronic Hand Eczema Using a Dedicated Case Report Form: A SIDAPA Multicentre Study
Source: Contact Dermatitis. 2026 Feb 15;94(6):662–76. doi: 10.1111/cod.70105 (PMC13139691; doi:10.1111/cod.70105)
Supplement: Supplementary file 1 — Table S1: Case Report Form (CRF) specifically designed for CHE patients. Table S2: Questionnaire to evaluate the usefulness and ease of use of the Case Report Form (CRF). [file COD-94-662-s001.docx]

**Supplementary Table 1.** Case Report Form (CRF) specifically designed for CHE patients

**__________________________________________________________________________________________________**

**Criteria for inclusion in the SIDAPA database of Chronic Hand Eczema (CHE)**

*If even one of the following criteria is not met, it is not possible to proceed with the compilation"*

The patient: 🞎 has eczema on the hands lasting over 3 months or recurring two or more times in the last year

🞎 is > 18 years old

🞎 signed the informed consent form on ___ / ___ / ______

🞎 has no mycosis or psoriasis of the hands

**Patient referred for hand eczema by:** 🞎 general practitioner 🞎 allergologist 🞎 dermatologist

🞎 occupational doctor 🞎 another medical specialist 🞎 none of the former (first consultation for HE)

**Patient initials (first and last name)** ____________ **Date of first visit** ___ / ___ / ______

**Birth date** ___ / ___ / ______ **Sex** 🞎 F 🞎 M **Weight** (kg) _____ **Height** (cm) _____

**Smoker**  🞎 yes 🞎 no 🞎 in the past

**Family history of atopy:** 🞎 yes 🞎 no 🞎 doubtful

**Personal history of atopy:**

Atopic dermatitis 🞎 yes, current 🞎 yes, past 🞎 no, never 🞎 doubtful

Allergic oculo-rhinitis 🞎 yes, current 🞎 yes, past 🞎 no, never 🞎 doubtful

Allergic asthma 🞎 yes, current 🞎 yes, past 🞎 no, never 🞎 doubtful

Known sensitization to aeroallergens 🞎 yes 🞎 no

Known sensitization to food allergens 🞎 yes 🞎 no

Other, e.g. known allergy to hymenoptera or drugs 🞎 yes 🞎 no

**Family history of** **psoriasis:** 🞎 yes 🞎 no 🞎 doubtful

**Educational level:** 🞎 elementary school 🞎 lower secondary school 🞎 high school 🞎 university degree

**Occupation**

***(for codes and definitions, see table below)***

**Current occupation (code):** _____ **for** _____ **years and** _____ **months**

**Type** 🞎 wetwork 🞎 non-wetwork but at risk for CHE 🞎 job not at risk for CHE

**Previous occupation (code):** _____ **for** _____ **years and** _____ **months**

| **WETWORK OCCUPATIONS**  *Where workers have wet/damp hands for 2 or more hours per day and/or wash their hands > 20-25 times per day and/or wear occlusive gloves for total cumulative 2 hours per day or more*   1. Healthcare workers (nurses, nursing assistants, doctors, surgeons, other healthcare workers) 2. Hairdressers, beauticians 3. Food workers (cooks, bartenders, pastry chefs, food clerks, fishmongers, etc.) 4. Metalworkers who use cutting oils 5. Other wetworkers (e.g. fishermen, workers in animal farms,   greenhouses, stables, pet stores, etc.)   1. Housewives | **NON-WETWORK MANUAL OCCUPATIONS AT RISK FOR CHE**  *due to traumiterative activities and/or exposure to irritants and/or allergens*   1. Construction workers (bricklayers, tilers, flooring contractors, plumbers, painters, etc.) 2. Chemical industry workers 3. Farmers and laborers 4. Metalworkers, welders, etc. 5. Carpenters 6. Electronic industry workers 7. Assemblers of rubber artifacts, printed circuit boards, etc. 8. Mechanics, coachbuilders, tire dealers, etc. 9. Other manual workers |
| --- | --- |
| **OTHER OCCUPATIONS**   1. Office workers (of all kinds) 2. Managers (of all types) 3. Teachers 4. Students 5. Retirees | 1. Healthcare professionals not devoted to wetwork 2. Computer technicians and other technicians 3. Merchants and vendors (not in wetwork categories) 4. Others (e.g. craftsmen not in contact with irritants, military personnel,   entertainment workers, sports workers, etc.) |

**Onset/worsening of hand eczema at work** 🞎 yes 🞎 no 🞎 doubtful

**Workdays lost due to hand eczema in the last year:**

🞎 none 🞎 <7 days 🞎 7-21 days 🞎 > 21 days 🞎 not applicable

**Patch test (every enrolled patient must undergo patch testing, even if already performed in the past)**

**Has the patient already undergone previous patch tests?** 🞎 no 🞎 yes, date ___ / ___ / ______ **Result 🞎 negative 🞎 positive (fill in the table below)**

|  |  | | | |  | | | | ***Exposure info, to be filled in only for relevant reactions*** | | | | | | | |
| --- | --- | --- | --- | --- | --- | --- | --- | --- | --- | --- | --- | --- | --- | --- | --- | --- |
| **Allergens** | **Positive reactions** | | | | **Relevance** | | | | **Contact Avoided?** | | | **Avoidable?** | | **Occupational?** | | |
| 1. Fragrance mix II 14% pet. | 🞎 + | 🞎 ++ | | 🞎 +++ | 🞎 current | 🞎 past | 🞎 doubtful | 🞎 no | 🞎 yes | 🞎 no | 🞎 doubtful | 🞎 yes | 🞎 no | 🞎 yes | 🞎 no | 🞎 partly |
| 1. Thiuram mix 1% pet. | 🞎 + | 🞎 ++ | | 🞎 +++ | 🞎 current | 🞎 past | 🞎 doubtful | 🞎 no | 🞎 yes | 🞎 no | 🞎 doubtful | 🞎 yes | 🞎 no | 🞎 yes | 🞎 no | 🞎 partly |
| 1. Potassium dichromate 0.5% pet. | 🞎 + | 🞎 ++ | | 🞎 +++ | 🞎 current | 🞎 past | 🞎 doubtful | 🞎 no | 🞎 yes | 🞎 no | 🞎 doubtful | 🞎 yes | 🞎 no | 🞎 yes | 🞎 no | 🞎 partly |
| 1. *Myroxylon pereirae* (Balsam of Peru) 25% pet. | 🞎 + | 🞎 ++ | | 🞎 +++ | 🞎 current | 🞎 past | 🞎 doubtful | 🞎 no | 🞎 yes | 🞎 no | 🞎 doubtful | 🞎 yes | 🞎 no | 🞎 yes | 🞎 no | 🞎 partly |
| 1. N-Isopropyl-N'-phenyl-p-phenylendiamine 0.1% pet. | 🞎 + | 🞎 ++ | | 🞎 +++ | 🞎 current | 🞎 past | 🞎 doubtful | 🞎 no | 🞎 yes | 🞎 no | 🞎 doubtful | 🞎 yes | 🞎 no | 🞎 yes | 🞎 no | 🞎 partly |
| 1. Methylisothiazolinone/methylchloroisothiazolinone 0.02% aq. | 🞎 + | 🞎 ++ | | 🞎 +++ | 🞎 current | 🞎 past | 🞎 doubtful | 🞎 no | 🞎 yes | 🞎 no | 🞎 doubtful | 🞎 yes | 🞎 no | 🞎 yes | 🞎 no | 🞎 partly |
| 1. p-Phenylendiamine 1% pet. | 🞎 + | 🞎 ++ | | 🞎 +++ | 🞎 current | 🞎 past | 🞎 doubtful | 🞎 no | 🞎 yes | 🞎 no | 🞎 doubtful | 🞎 yes | 🞎 no | 🞎 yes | 🞎 no | 🞎 partly |
| 1. Lanolin alcohol 30% pet. | 🞎 + | 🞎 ++ | | 🞎 +++ | 🞎 current | 🞎 past | 🞎 doubtful | 🞎 no | 🞎 yes | 🞎 no | 🞎 doubtful | 🞎 yes | 🞎 no | 🞎 yes | 🞎 no | 🞎 partly |
| 1. Colophony 20% pet. | 🞎 + | 🞎 ++ | | 🞎 +++ | 🞎 current | 🞎 past | 🞎 doubtful | 🞎 no | 🞎 yes | 🞎 no | 🞎 doubtful | 🞎 yes | 🞎 no | 🞎 yes | 🞎 no | 🞎 partly |
| 1. Neomycin sulphate 20% pet. | 🞎 + | 🞎 ++ | | 🞎 +++ | 🞎 current | 🞎 past | 🞎 doubtful | 🞎 no | 🞎 yes | 🞎 no | 🞎 doubtful | 🞎 yes | 🞎 no | 🞎 yes | 🞎 no | 🞎 partly |
| 1. Mercaptobenzothiazole mix 2% pet. | 🞎 + | 🞎 ++ | | 🞎 +++ | 🞎 current | 🞎 past | 🞎 doubtful | 🞎 no | 🞎 yes | 🞎 no | 🞎 doubtful | 🞎 yes | 🞎 no | 🞎 yes | 🞎 no | 🞎 partly |
| 1. Epoxy resin 1% pet. | 🞎 + | 🞎 ++ | | 🞎 +++ | 🞎 current | 🞎 past | 🞎 doubtful | 🞎 no | 🞎 yes | 🞎 no | 🞎 doubtful | 🞎 yes | 🞎 no | 🞎 yes | 🞎 no | 🞎 partly |
| 1. Formaldehyde 2% aq. | 🞎 + | 🞎 ++ | | 🞎 +++ | 🞎 current | 🞎 past | 🞎 doubtful | 🞎 no | 🞎 yes | 🞎 no | 🞎 doubtful | 🞎 yes | 🞎 no | 🞎 yes | 🞎 no | 🞎 partly |
| 1. 2-Mercaptobenzothiazole 2% pet. | 🞎 + | 🞎 ++ | | 🞎 +++ | 🞎 current | 🞎 past | 🞎 doubtful | 🞎 no | 🞎 yes | 🞎 no | 🞎 doubtful | 🞎 yes | 🞎 no | 🞎 yes | 🞎 no | 🞎 partly |
| 1. p-ter-Butyl-phenol-formaldehyde resin 1% pet. | 🞎 + | 🞎 ++ | | 🞎 +++ | 🞎 current | 🞎 past | 🞎 doubtful | 🞎 no | 🞎 yes | 🞎 no | 🞎 doubtful | 🞎 yes | 🞎 no | 🞎 yes | 🞎 no | 🞎 partly |
| 1. Nickel sulfate hexahydrate 5% pet. | 🞎 + | 🞎 ++ | | 🞎 +++ | 🞎 current | 🞎 past | 🞎 doubtful | 🞎 no | 🞎 yes | 🞎 no | 🞎 doubtful | 🞎 yes | 🞎 no | 🞎 yes | 🞎 no | 🞎 partly |
| 1. Fragrance mix I 8% + sorbitan sesquioleate 5% pet. | 🞎 + | 🞎 ++ | | 🞎 +++ | 🞎 current | 🞎 past | 🞎 doubtful | 🞎 no | 🞎 yes | 🞎 no | 🞎 doubtful | 🞎 yes | 🞎 no | 🞎 yes | 🞎 no | 🞎 partly |
| 1. Disperse dye mix 6.6% pet. | 🞎 + | 🞎 ++ | | 🞎 +++ | 🞎 current | 🞎 past | 🞎 doubtful | 🞎 no | 🞎 yes | 🞎 no | 🞎 doubtful | 🞎 yes | 🞎 no | 🞎 yes | 🞎 no | 🞎 partly |
| 1. Parabens mix 16% pet. | 🞎 + | 🞎 ++ | | 🞎 +++ | 🞎 current | 🞎 past | 🞎 doubtful | 🞎 no | 🞎 yes | 🞎 no | 🞎 doubtful | 🞎 yes | 🞎 no | 🞎 yes | 🞎 no | 🞎 partly |
| 1. Cobalt chloride hexahydrate 1% pet. | 🞎 + | 🞎 ++ | | 🞎 +++ | 🞎 current | 🞎 past | 🞎 doubtful | 🞎 no | 🞎 yes | 🞎 no | 🞎 doubtful | 🞎 yes | 🞎 no | 🞎 yes | 🞎 no | 🞎 partly |
| 1. 3-Dimethylamino-1-propylamine 1% aq. | 🞎 + | 🞎 ++ | | 🞎 +++ | 🞎 current | 🞎 past | 🞎 doubtful | 🞎 no | 🞎 yes | 🞎 no | 🞎 doubtful | 🞎 yes | 🞎 no | 🞎 yes | 🞎 no | 🞎 partly |
| 1. Budesonide 0.01% pet. | 🞎 + | 🞎 ++ | | 🞎 +++ | 🞎 current | 🞎 past | 🞎 doubtful | 🞎 no | 🞎 yes | 🞎 no | 🞎 doubtful | 🞎 yes | 🞎 no | 🞎 yes | 🞎 no | 🞎 partly |
| 1. Hydroxymethylpentylcyclohexenecarboxaldehyde (Lyral) 5% pet. | 🞎 + | 🞎 ++ | | 🞎 +++ | 🞎 current | 🞎 past | 🞎 doubtful | 🞎 no | 🞎 yes | 🞎 no | 🞎 doubtful | 🞎 yes | 🞎 no | 🞎 yes | 🞎 no | 🞎 partly |
| 1. 2-Methyl-4-isothiazolin-3-one 0.2% aq. | 🞎 + | 🞎 ++ | | 🞎 +++ | 🞎 current | 🞎 past | 🞎 doubtful | 🞎 no | 🞎 yes | 🞎 no | 🞎 doubtful | 🞎 yes | 🞎 no | 🞎 yes | 🞎 no | 🞎 partly |
| 1. Sorbitan sesquioleate 20% pet. | 🞎 + | 🞎 ++ | | 🞎 +++ | 🞎 current | 🞎 past | 🞎 doubtful | 🞎 no | 🞎 yes | 🞎 no | 🞎 doubtful | 🞎 yes | 🞎 no | 🞎 yes | 🞎 no | 🞎 partly |
| 1. 2-Hydroxyethylmetacrylate 1% pet. | 🞎 + | 🞎 ++ | | 🞎 +++ | 🞎 current | 🞎 past | 🞎 doubtful | 🞎 no | 🞎 yes | 🞎 no | 🞎 doubtful | 🞎 yes | 🞎 no | 🞎 yes | 🞎 no | 🞎 partly |
| 1. Caine mix 10% pet. | 🞎 + | 🞎 ++ | | 🞎 +++ | 🞎 current | 🞎 past | 🞎 doubtful | 🞎 no | 🞎 yes | 🞎 no | 🞎 doubtful | 🞎 yes | 🞎 no | 🞎 yes | 🞎 no | 🞎 partly |
| 1. Tixocortol pivalate 1% pet. | 🞎 + | 🞎 ++ | | 🞎 +++ | 🞎 current | 🞎 past | 🞎 doubtful | 🞎 no | 🞎 yes | 🞎 no | 🞎 doubtful | 🞎 yes | 🞎 no | 🞎 yes | 🞎 no | 🞎 partly |
| 1. Cocamidopropylbetaine 1% pet. | 🞎 + | 🞎 ++ | | 🞎 +++ | 🞎 current | 🞎 past | 🞎 doubtful | 🞎 no | 🞎 yes | 🞎 no | 🞎 doubtful | 🞎 yes | 🞎 no | 🞎 yes | 🞎 no | 🞎 partly |
| 1. Sodium metabisulphite 1% pet. | 🞎 + | 🞎 ++ | | 🞎 +++ | 🞎 current | 🞎 past | 🞎 doubtful | 🞎 no | 🞎 yes | 🞎 no | 🞎 doubtful | 🞎 yes | 🞎 no | 🞎 yes | 🞎 no | 🞎 partly |
| 1. Benzisothiazolinone 0.1% aq. | 🞎 + | 🞎 ++ | | 🞎 +++ | 🞎 current | 🞎 past | 🞎 doubtful | 🞎 no | 🞎 yes | 🞎 no | 🞎 doubtful | 🞎 yes | 🞎 no | 🞎 yes | 🞎 no | 🞎 partly |
| 1. Compositae mix II 6% pet. | 🞎 + | 🞎 ++ | | 🞎 +++ | 🞎 current | 🞎 past | 🞎 doubtful | 🞎 no | 🞎 yes | 🞎 no | 🞎 doubtful | 🞎 yes | 🞎 no | 🞎 yes | 🞎 no | 🞎 partly |
| 1. OTHERS | 🞎 yes | | 🞎 no | |  |  |  |  |  |  |  |  |  | 🞎 yes | 🞎 no | 🞎 partly |

### Current patch tests (performed at enrolment in the study)

**Date** ___ / ___ / ______  **Result 🞎 negative 🞎 positive (fill in the table below)**

|  |  | | | |  | | | | ***Exposure info, to be filled in only for relevant reactions*** | | | | | | | |
| --- | --- | --- | --- | --- | --- | --- | --- | --- | --- | --- | --- | --- | --- | --- | --- | --- |
| **Allergens** | **Positive reactions** | | | | **Relevance** | | | | **Contact Avoided?** | | | **Avoidable?** | | **Occupational?** | | |
| 1. Fragrance mix II 14% pet. | 🞎 + | 🞎 ++ | | 🞎 +++ | 🞎 current | 🞎 past | 🞎 doubtful | 🞎 no | 🞎 yes | 🞎 no | 🞎 doubtful | 🞎 yes | 🞎 no | 🞎 yes | 🞎 no | 🞎 partly |
| 1. Thiuram mix 1% pet. | 🞎 + | 🞎 ++ | | 🞎 +++ | 🞎 current | 🞎 past | 🞎 doubtful | 🞎 no | 🞎 yes | 🞎 no | 🞎 doubtful | 🞎 yes | 🞎 no | 🞎 yes | 🞎 no | 🞎 partly |
| 1. Potassium dichromate 0.5% pet. | 🞎 + | 🞎 ++ | | 🞎 +++ | 🞎 current | 🞎 past | 🞎 doubtful | 🞎 no | 🞎 yes | 🞎 no | 🞎 doubtful | 🞎 yes | 🞎 no | 🞎 yes | 🞎 no | 🞎 partly |
| 1. *Myroxylon pereirae* (Balsam of Peru) 25% pet. | 🞎 + | 🞎 ++ | | 🞎 +++ | 🞎 current | 🞎 past | 🞎 doubtful | 🞎 no | 🞎 yes | 🞎 no | 🞎 doubtful | 🞎 yes | 🞎 no | 🞎 yes | 🞎 no | 🞎 partly |
| 1. N-Isopropyl-N'-phenyl-p-phenylendiamine 0.1% pet. | 🞎 + | 🞎 ++ | | 🞎 +++ | 🞎 current | 🞎 past | 🞎 doubtful | 🞎 no | 🞎 yes | 🞎 no | 🞎 doubtful | 🞎 yes | 🞎 no | 🞎 yes | 🞎 no | 🞎 partly |
| 1. Methylisothiazolinone/methylchloroisothiazolinone 0.02% aq. | 🞎 + | 🞎 ++ | | 🞎 +++ | 🞎 current | 🞎 past | 🞎 doubtful | 🞎 no | 🞎 yes | 🞎 no | 🞎 doubtful | 🞎 yes | 🞎 no | 🞎 yes | 🞎 no | 🞎 partly |
| 1. p-Phenylendiamine 1% pet. | 🞎 + | 🞎 ++ | | 🞎 +++ | 🞎 current | 🞎 past | 🞎 doubtful | 🞎 no | 🞎 yes | 🞎 no | 🞎 doubtful | 🞎 yes | 🞎 no | 🞎 yes | 🞎 no | 🞎 partly |
| 1. Lanolin alcohol 30% pet. | 🞎 + | 🞎 ++ | | 🞎 +++ | 🞎 current | 🞎 past | 🞎 doubtful | 🞎 no | 🞎 yes | 🞎 no | 🞎 doubtful | 🞎 yes | 🞎 no | 🞎 yes | 🞎 no | 🞎 partly |
| 1. Colophony 20% pet. | 🞎 + | 🞎 ++ | | 🞎 +++ | 🞎 current | 🞎 past | 🞎 doubtful | 🞎 no | 🞎 yes | 🞎 no | 🞎 doubtful | 🞎 yes | 🞎 no | 🞎 yes | 🞎 no | 🞎 partly |
| 1. Neomycin sulphate 20% pet. | 🞎 + | 🞎 ++ | | 🞎 +++ | 🞎 current | 🞎 past | 🞎 doubtful | 🞎 no | 🞎 yes | 🞎 no | 🞎 doubtful | 🞎 yes | 🞎 no | 🞎 yes | 🞎 no | 🞎 partly |
| 1. Mercaptobenzothiazole mix 2% pet. | 🞎 + | 🞎 ++ | | 🞎 +++ | 🞎 current | 🞎 past | 🞎 doubtful | 🞎 no | 🞎 yes | 🞎 no | 🞎 doubtful | 🞎 yes | 🞎 no | 🞎 yes | 🞎 no | 🞎 partly |
| 1. Epoxy resin 1% pet. | 🞎 + | 🞎 ++ | | 🞎 +++ | 🞎 current | 🞎 past | 🞎 doubtful | 🞎 no | 🞎 yes | 🞎 no | 🞎 doubtful | 🞎 yes | 🞎 no | 🞎 yes | 🞎 no | 🞎 partly |
| 1. Formaldehyde 2% aq. | 🞎 + | 🞎 ++ | | 🞎 +++ | 🞎 current | 🞎 past | 🞎 doubtful | 🞎 no | 🞎 yes | 🞎 no | 🞎 doubtful | 🞎 yes | 🞎 no | 🞎 yes | 🞎 no | 🞎 partly |
| 1. 2-Mercaptobenzothiazole 2% pet. | 🞎 + | 🞎 ++ | | 🞎 +++ | 🞎 current | 🞎 past | 🞎 doubtful | 🞎 no | 🞎 yes | 🞎 no | 🞎 doubtful | 🞎 yes | 🞎 no | 🞎 yes | 🞎 no | 🞎 partly |
| 1. p-ter-Butyl-phenol-formaldehyde resin 1% pet. | 🞎 + | 🞎 ++ | | 🞎 +++ | 🞎 current | 🞎 past | 🞎 doubtful | 🞎 no | 🞎 yes | 🞎 no | 🞎 doubtful | 🞎 yes | 🞎 no | 🞎 yes | 🞎 no | 🞎 partly |
| 1. Nickel sulfate hexahydrate 5% pet. | 🞎 + | 🞎 ++ | | 🞎 +++ | 🞎 current | 🞎 past | 🞎 doubtful | 🞎 no | 🞎 yes | 🞎 no | 🞎 doubtful | 🞎 yes | 🞎 no | 🞎 yes | 🞎 no | 🞎 partly |
| 1. Fragrance mix I 8% + sorbitan sesquioleate 5% pet. | 🞎 + | 🞎 ++ | | 🞎 +++ | 🞎 current | 🞎 past | 🞎 doubtful | 🞎 no | 🞎 yes | 🞎 no | 🞎 doubtful | 🞎 yes | 🞎 no | 🞎 yes | 🞎 no | 🞎 partly |
| 1. Disperse dye mix 6.6% pet. | 🞎 + | 🞎 ++ | | 🞎 +++ | 🞎 current | 🞎 past | 🞎 doubtful | 🞎 no | 🞎 yes | 🞎 no | 🞎 doubtful | 🞎 yes | 🞎 no | 🞎 yes | 🞎 no | 🞎 partly |
| 1. Parabens mix 16% pet. | 🞎 + | 🞎 ++ | | 🞎 +++ | 🞎 current | 🞎 past | 🞎 doubtful | 🞎 no | 🞎 yes | 🞎 no | 🞎 doubtful | 🞎 yes | 🞎 no | 🞎 yes | 🞎 no | 🞎 partly |
| 1. Cobalt chloride hexahydrate 1% pet. | 🞎 + | 🞎 ++ | | 🞎 +++ | 🞎 current | 🞎 past | 🞎 doubtful | 🞎 no | 🞎 yes | 🞎 no | 🞎 doubtful | 🞎 yes | 🞎 no | 🞎 yes | 🞎 no | 🞎 partly |
| 1. 3-Dimethylamino-1-propylamine 1% aq. | 🞎 + | 🞎 ++ | | 🞎 +++ | 🞎 current | 🞎 past | 🞎 doubtful | 🞎 no | 🞎 yes | 🞎 no | 🞎 doubtful | 🞎 yes | 🞎 no | 🞎 yes | 🞎 no | 🞎 partly |
| 1. Budesonide 0.01% pet. | 🞎 + | 🞎 ++ | | 🞎 +++ | 🞎 current | 🞎 past | 🞎 doubtful | 🞎 no | 🞎 yes | 🞎 no | 🞎 doubtful | 🞎 yes | 🞎 no | 🞎 yes | 🞎 no | 🞎 partly |
| 1. Hydroxymethylpentylcyclohexenecarboxaldehyde (Lyral) 5% pet. | 🞎 + | 🞎 ++ | | 🞎 +++ | 🞎 current | 🞎 past | 🞎 doubtful | 🞎 no | 🞎 yes | 🞎 no | 🞎 doubtful | 🞎 yes | 🞎 no | 🞎 yes | 🞎 no | 🞎 partly |
| 1. 2-Methyl-4-isothiazolin-3-one 0.2% aq. | 🞎 + | 🞎 ++ | | 🞎 +++ | 🞎 current | 🞎 past | 🞎 doubtful | 🞎 no | 🞎 yes | 🞎 no | 🞎 doubtful | 🞎 yes | 🞎 no | 🞎 yes | 🞎 no | 🞎 partly |
| 1. Sorbitan sesquioleate 20% pet. | 🞎 + | 🞎 ++ | | 🞎 +++ | 🞎 current | 🞎 past | 🞎 doubtful | 🞎 no | 🞎 yes | 🞎 no | 🞎 doubtful | 🞎 yes | 🞎 no | 🞎 yes | 🞎 no | 🞎 partly |
| 1. 2-Hydroxyethylmetacrylate 1% pet. | 🞎 + | 🞎 ++ | | 🞎 +++ | 🞎 current | 🞎 past | 🞎 doubtful | 🞎 no | 🞎 yes | 🞎 no | 🞎 doubtful | 🞎 yes | 🞎 no | 🞎 yes | 🞎 no | 🞎 partly |
| 1. Caine mix 10% pet. | 🞎 + | 🞎 ++ | | 🞎 +++ | 🞎 current | 🞎 past | 🞎 doubtful | 🞎 no | 🞎 yes | 🞎 no | 🞎 doubtful | 🞎 yes | 🞎 no | 🞎 yes | 🞎 no | 🞎 partly |
| 1. Tixocortol pivalate 1% pet. | 🞎 + | 🞎 ++ | | 🞎 +++ | 🞎 current | 🞎 past | 🞎 doubtful | 🞎 no | 🞎 yes | 🞎 no | 🞎 doubtful | 🞎 yes | 🞎 no | 🞎 yes | 🞎 no | 🞎 partly |
| 1. Cocamidopropylbetaine 1% pet. | 🞎 + | 🞎 ++ | | 🞎 +++ | 🞎 current | 🞎 past | 🞎 doubtful | 🞎 no | 🞎 yes | 🞎 no | 🞎 doubtful | 🞎 yes | 🞎 no | 🞎 yes | 🞎 no | 🞎 partly |
| 1. Sodium metabisulphite 1% pet. | 🞎 + | 🞎 ++ | | 🞎 +++ | 🞎 current | 🞎 past | 🞎 doubtful | 🞎 no | 🞎 yes | 🞎 no | 🞎 doubtful | 🞎 yes | 🞎 no | 🞎 yes | 🞎 no | 🞎 partly |
| 1. Benzisothiazolinone 0.1% aq. | 🞎 + | 🞎 ++ | | 🞎 +++ | 🞎 current | 🞎 past | 🞎 doubtful | 🞎 no | 🞎 yes | 🞎 no | 🞎 doubtful | 🞎 yes | 🞎 no | 🞎 yes | 🞎 no | 🞎 partly |
| 1. Compositae mix II 6% pet. | 🞎 + | 🞎 ++ | | 🞎 +++ | 🞎 current | 🞎 past | 🞎 doubtful | 🞎 no | 🞎 yes | 🞎 no | 🞎 doubtful | 🞎 yes | 🞎 no | 🞎 yes | 🞎 no | 🞎 partly |
| 1. OTHERS | 🞎 yes | | 🞎 no | |  |  |  |  |  |  |  |  |  | 🞎 yes | 🞎 no | 🞎 partly |

### Other exogenous risk factors

Habitual manual leisure activities 🞎 gardening 🞎 do it yourself 🞎 other

The patient has taken/takes care of small children (<3 years)? 🞎 no 🞎 yes

Housework/cooking?  🞎 never 🞎 sometime 🞎 every day

Hand washing frequency (times per day)? 🞎 0-10 🞎 11-20 🞎 > 20

Use of sanitizing gels?  🞎 never 🞎 sometime 🞎 1-5 times/day

🞎 5-10 times/day 🞎 10-20 times/day 🞎 >20 times/day

Use of hand cream? (*protective factor*) 🞎 never 🞎 sometime 🞎 once a day

🞎 twice a day 🞎 3 times/day 🞎 >3 times/day

**Clinical course, morphology, and classification**

**When did the patient’s HE start? (indicate the approximate year)** _____

**If HE is recurrent, how many episodes have there been in the last year?** _____

**How many months ago did the current episode begin?** _____

## What is the course of the disease?

🞎 it comes and goes 🞎 always present, never heals completely 🞎 it has gotten worse over time

**Localization:** See HEES scoring on the next page

**Morphology:** See HECSI scoring on the next page

**Classification (one or more of the following)**

🞎 ACD 🞎 ICD 🞎 Atopic dermatitis of the hands 🞎 Pulpitis

🞎 Nummular HE 🞎 Hypercheratotic palmar eczema

🞎 Acute recurrent vesicular HE 🞎 Contact Urticaria / Protein Contact Dermatitis

**Is HE associated with dermatitis in other body areas?**

🞎 no 🞎 soles 🞎 face 🞎 limbs 🞎 trunk 🞎 widespread

**Photographs (mandatory, upload online)**

Take photographs of the palmar and dorsal surface of both hands (with fingers spread) and wrists

🞎 done

🞎 no photo because the patient has denied permission

**Past and current treatments** (select all applicable options)

**Low-to-medium potency topical corticosteroids**

🞎 current 🞎 past 🞎 both current and past 🞎 no 🞎 unknown

If yes, with benefit? 🞎 yes 🞎 no 🞎 transitory 🞎 worsening

🞎 Treatment stopped due to side effects

**High potency topical corticosteroids**

🞎 current 🞎 past 🞎 both current and past 🞎 no 🞎 unknown

If yes, with benefit? 🞎 yes 🞎 no 🞎 transitory 🞎 worsening

🞎 Treatment stopped due to side effects

**Pimecrolimus**

🞎 current 🞎 past 🞎 both current and past 🞎 no 🞎 unknown

If yes, with benefit? 🞎 yes 🞎 no 🞎 transitory 🞎 worsening

🞎 Treatment stopped due to side effects

**Tacrolimus**

🞎 current 🞎 past 🞎 both current and past 🞎 no 🞎 unknown

If yes, with benefit? 🞎 yes 🞎 no 🞎 transitory 🞎 worsening

🞎 Treatment stopped due to side effects

**Oral corticosteroids**

🞎 current 🞎 past 🞎 both current and past 🞎 no 🞎 unknown

If yes, with benefit? 🞎 yes 🞎 no 🞎 transitory 🞎 worsening

🞎 Treatment stopped due to side effects

**Alitretinoin**

🞎 current 🞎 past 🞎 both current and past 🞎 no 🞎 unknown

If yes, with benefit? 🞎 yes 🞎 no 🞎 transitory 🞎 worsening

🞎 Treatment stopped due to side effects

**Cyclosporin**

🞎 current 🞎 past 🞎 both current and past 🞎 no 🞎 unknown

If yes, with benefit? 🞎 yes 🞎 no 🞎 transitory 🞎 worsening

🞎 Treatment stopped due to side effects

**Other systemic immunosuppressants (methotrexate, azathioprine…)**

🞎 current 🞎 past 🞎 both current and past 🞎 no 🞎 unknown

If yes, with benefit? 🞎 yes 🞎 no 🞎 transitory 🞎 worsening

🞎 Treatment stopped due to side effects

**Dupilumab**

🞎 current 🞎 past 🞎 both current and past 🞎 no 🞎 unknown

If yes, with benefit? 🞎 yes 🞎 no 🞎 transitory 🞎 worsening

🞎 Treatment stopped due to side effects

**Other biologics**

🞎 current 🞎 past 🞎 both current and past 🞎 no 🞎 unknown

If yes, with benefit? 🞎 yes 🞎 no 🞎 transitory 🞎 worsening

🞎 Treatment stopped due to side effects

**Phototherapy**

🞎 current 🞎 past 🞎 both current and past 🞎 no 🞎 unknown

If yes, with benefit? 🞎 yes 🞎 no 🞎 transitory 🞎 worsening

🞎 Treatment stopped due to side effects

**Use of topical non-pharmacological agents** (select all applicable options)

**Emollients** 🞎 Basic hand cream 🞎 Emollient with antinflammatory or barrier repair formulation 🞎 None

**Detergents** 🞎 Cleansing oil/cream 🞎 Foaming detergent/soap

**HEES (Hand Eczema Extent Score)^22^**

## Right hand

Back of hand 🞎 more than 2/3 🞎 less than 2/3

Palm 🞎 more than 2/3 🞎 less than 2/3

**Finger Back of finger Side of finger Underside of finger Finger tip Nail changes**

Thumb 🞎 🞎 🞎 🞎 🞎

Index finger 🞎 🞎 🞎 🞎 🞎

Middle finger 🞎 🞎 🞎 🞎 🞎

Ring finger 🞎 🞎 🞎 🞎 🞎

Little finger 🞎 🞎 🞎 🞎 🞎

**Finger-web between…** 🞎 thumb & index finger 🞎 index finger & middle finger
 🞎 middle finger & ring finger 🞎 ring finger & little finger

## Left hand

Back of hand 🞎 more than 2/3 🞎 less than 2/3

Palm 🞎 more than 2/3 🞎 less than 2/3

**Finger Back of finger Side of finger Underside of finger Finger tip Nail changes**

Thumb 🞎 🞎 🞎 🞎 🞎

Index finger 🞎 🞎 🞎 🞎 🞎

Middle finger 🞎 🞎 🞎 🞎 🞎

Ring finger 🞎 🞎 🞎 🞎 🞎

Little finger 🞎 🞎 🞎 🞎 🞎

**Finger-web between…** 🞎 thumb & index finger 🞎 index finger & middle finger
 🞎 middle finger & ring finger 🞎 ring finger & little finger

**HECSI (Hand ECzema Severity Index)^23^**

| Fingertips | Erythema 🞎 0 🞎 1 🞎 2 🞎 3 Infiltration ⁄papulation 🞎 0 🞎 1 🞎 2 🞎 3  Vesicles 🞎 0 🞎 1 🞎 2 🞎 3  Fissures 🞎 0 🞎 1 🞎 2 🞎 3  Scaling 🞎 0 🞎 1 🞎 2 🞎 3  Oedema 🞎 0 🞎 1 🞎 2 🞎 3  Area score 🞎 0% 🞎 1-25% 🞎 26-50% 🞎 51-75% 🞎 76-100% |
| --- | --- |
| Fingers  (except tips) | Erythema 🞎 0 🞎 1 🞎 2 🞎 3 Infiltration ⁄papulation 🞎 0 🞎 1 🞎 2 🞎 3  Vesicles 🞎 0 🞎 1 🞎 2 🞎 3  Fissures 🞎 0 🞎 1 🞎 2 🞎 3  Scaling 🞎 0 🞎 1 🞎 2 🞎 3  Oedema 🞎 0 🞎 1 🞎 2 🞎 3  Area score 🞎 0% 🞎 1-25% 🞎 26-50% 🞎 51-75% 🞎 76-100% |
| Palm of hands | Erythema 🞎 0 🞎 1 🞎 2 🞎 3 Infiltration ⁄papulation 🞎 0 🞎 1 🞎 2 🞎 3  Vesicles 🞎 0 🞎 1 🞎 2 🞎 3  Fissures 🞎 0 🞎 1 🞎 2 🞎 3  Scaling 🞎 0 🞎 1 🞎 2 🞎 3  Oedema 🞎 0 🞎 1 🞎 2 🞎 3  Area score 🞎 0% 🞎 1-25% 🞎 26-50% 🞎 51-75% 🞎 76-100% |
| Back of hands | Erythema 🞎 0 🞎 1 🞎 2 🞎 3 Infiltration ⁄papulation 🞎 0 🞎 1 🞎 2 🞎 3  Vesicles 🞎 0 🞎 1 🞎 2 🞎 3  Fissures 🞎 0 🞎 1 🞎 2 🞎 3  Scaling 🞎 0 🞎 1 🞎 2 🞎 3  Oedema 🞎 0 🞎 1 🞎 2 🞎 3  Area score 🞎 0% 🞎 1-25% 🞎 26-50% 🞎 51-75% 🞎 76-100% |
| Wrists | Erythema 🞎 0 🞎 1 🞎 2 🞎 3 Infiltration ⁄papulation 🞎 0 🞎 1 🞎 2 🞎 3  Vesicles 🞎 0 🞎 1 🞎 2 🞎 3  Fissures 🞎 0 🞎 1 🞎 2 🞎 3  Scaling 🞎 0 🞎 1 🞎 2 🞎 3  Oedema 🞎 0 🞎 1 🞎 2 🞎 3  Area score 🞎 0% 🞎 1-25% 🞎 26-50% 🞎 51-75% 🞎 76-100% |

# IGA-CHE (Investigator's Global Assessment-Chronic Hand Eczema)^24^

# Current severity of hand eczema

# 🞎 0 (clear) 🞎 1 (almost clear) 🞎 2 (mild) 🞎 3 (moderate) 🞎 4 (severe)

# PaGA-CHE (Patient's Global Assessment of CHE)^25^

How would you rate severity of your hand eczema at the moment?

# 🞎 0 (clear) 🞎 1 (almost clear) 🞎 2 (mild) 🞎 3 (moderate) 🞎 4 (severe)

## NRS-itch (Numerical Rating Scale of itch)^26^

How severe was the itch caused by your hand eczema in the last 7 days, on a scale from 0 (no itch) to 10 (worst imaginable itch)? _____

## NRS-pain (Numerical Rating Scale of pain)^27^

How severe was the pain caused by your hand eczema in the last 7 days, on a scale from 0 (no pain) to 10 (worst imaginable pain)? _____

# DLQI (Dermatology Life Quality Index)^30^

The aim of this questionnaire is to measure how much your skin problem has affected your life

over the last week. Please tick one box for each question.

| 1. | Over the last week, how **itchy**, **sore**, **painful** or **stinging** has your skin been? | Very much  A lot  A little  Not at all | 🞎 🞎 🞎 🞎 |  |
| --- | --- | --- | --- | --- |
| 2. | Over the last week, how **embarrassed** or **self conscious** have you been because of your skin? | Very much  A lot  A little  Not at all | 🞎 🞎 🞎 🞎 |  |
| 3. | Over the last week, how much has your skin interfered with you going **shopping** or looking after your **home** or **garden**? | Very much  A lot  A little  Not at all | 🞎 🞎 🞎 🞎 | Not relevant 🞎 |
| 4. | Over the last week, how much has your skin influenced the **clothes** you wear? | Very much  A lot  A little  Not at all | 🞎 🞎 🞎 🞎 | Not relevant 🞎 |
| 5. | Over the last week, how much has your skin affected any **social** or **leisure** activities? | Very much  A lot  A little  Not at all | 🞎 🞎 🞎 🞎 | Not relevant 🞎 |
| 6. | Over the last week, how much has your skin made it difficult for you to do any **sport**? | Very much  A lot  A little  Not at all | 🞎 🞎 🞎 🞎 | Not relevant 🞎 |
| 7. | Over the last week, has your skin prevented you from **working** or **studying**? | Yes  No | 🞎 🞎 | Not relevant 🞎 |
|  | If "No", over the last week how much has your skin been a problem at **work** or **studying**? | A lot  A little  Not at all | 🞎 🞎 🞎 |  |
| 8. | Over the last week, how much has your skin created problems with your **partner** or any of your **close friends** or **relatives**? | Very much  A lot  A little  Not at all | 🞎 🞎 🞎 🞎 | Not relevant 🞎 |
| 9. | Over the last week, how much has your skin caused any **sexual difficulties**? | Very much  A lot  A little  Not at all | 🞎 🞎 🞎 🞎 | Not relevant 🞎 |
| 10. | Over the last week, how much of a problem has the **treatment** for your skin been, for example by making your home messy, or by taking up time? | Very much  A lot  A little  Not at all | 🞎 🞎 🞎 🞎 | Not relevant 🞎 |

**QOLHEQ - Quality of Life in Hand Eczema Questionnaire^31,32^**

Please indicate how often you were bothered by the following situations during the last seven days:

|  | **I have been bothered by the skin condition of my hands…** | **never** | **rarely** | **sometimes** | **often** | **all the time** |
| --- | --- | --- | --- | --- | --- | --- |
| 1 | … being painful | 🞏 | 🞏 | 🞏 | 🞏 | 🞏 |
| 2 | … restricting/impairing me in my job | 🞏 | 🞏 | 🞏 | 🞏 | 🞏 |
| 3 | … restricting/impairing me in doing everyday home duties | 🞏 | 🞏 | 🞏 | 🞏 | 🞏 |
| 4 | … because I have to wear gloves | 🞏 | 🞏 | 🞏 | 🞏 | 🞏 |
| 5 | … making me feel frustrated | 🞏 | 🞏 | 🞏 | 🞏 | 🞏 |
| 6 | … itching | 🞏 | 🞏 | 🞏 | 🞏 | 🞏 |
| 7 | … because treatment is time consuming | 🞏 | 🞏 | 🞏 | 🞏 | 🞏 |
| 8 | … making me feel annoyed | 🞏 | 🞏 | 🞏 | 🞏 | 🞏 |
| 9 | … causing loss of sleep | 🞏 | 🞏 | 🞏 | 🞏 | 🞏 |
| 10 | … making me feel anxious about the future | 🞏 | 🞏 | 🞏 | 🞏 | 🞏 |
| 11 | … fissuring | 🞏 | 🞏 | 🞏 | 🞏 | 🞏 |
| 12 | … restricting/impairing me in my leisure time activities (e.g.   sports, hobbies) | 🞏 | 🞏 | 🞏 | 🞏 | 🞏 |
| 13 | … because I have to use creams | 🞏 | 🞏 | 🞏 | 🞏 | 🞏 |
| 14 | … causing problems washing myself | 🞏 | 🞏 | 🞏 | 🞏 | 🞏 |
| 15 | … causing problems dressing myself | 🞏 | 🞏 | 🞏 | 🞏 | 🞏 |
| 16 | … making me feel I have to hide my hands | 🞏 | 🞏 | 🞏 | 🞏 | 🞏 |
| 17 | … because it leads to me avoiding contact with other people | 🞏 | 🞏 | 🞏 | 🞏 | 🞏 |
| 18 | … because I have to visit a physician | 🞏 | 🞏 | 🞏 | 🞏 | 🞏 |
| 19 | … making me feel sad / depressed | 🞏 | 🞏 | 🞏 | 🞏 | 🞏 |
| 20 | … because of redness | 🞏 | 🞏 | 🞏 | 🞏 | 🞏 |
| 21 | … making me feel irritated | 🞏 | 🞏 | 🞏 | 🞏 | 🞏 |
| 22 | … because I have to avoid contact with certain things | 🞏 | 🞏 | 🞏 | 🞏 | 🞏 |
| 23 | … bleeding | 🞏 | 🞏 | 🞏 | 🞏 | 🞏 |
| 24 | … because of worrying about side effects of treatment | 🞏 | 🞏 | 🞏 | 🞏 | 🞏 |
| 25 | … affecting my family life and friendships | 🞏 | 🞏 | 🞏 | 🞏 | 🞏 |
| 26 | … because of the treatment costs I have to cover myself | 🞏 | 🞏 | 🞏 | 🞏 | 🞏 |
| 27 | … making me feel embarrassed | 🞏 | 🞏 | 🞏 | 🞏 | 🞏 |
| 28 | … because of dryness | 🞏 | 🞏 | 🞏 | 🞏 | 🞏 |
| 29 | … when touching my family or partner | 🞏 | 🞏 | 🞏 | 🞏 | 🞏 |
| 30 | … making me feel nervous | 🞏 | 🞏 | 🞏 | 🞏 | 🞏 |

**Supplementary Table 2.** Questionnaire to evaluate the usefulness and ease of use of the Case Report Form (CRF)

| *Please rate your degree of agreement with each of the following sentences with a number from 1 (total disagreement) to 5 (complete agreement).*   - The CRF allows for a comprehensive classification of patients with CHE - The CRF is intuitive and easy to fill in - The time required to fill in the CRF is compatible with clinical activity   How long does it take to complete the CRF for a patient? (number of minutes)  *Please indicate whether the following sections of the questionnaire are easy to fill in, with a score from 1 (I do not agree, it is difficult) to 5 (I totally agree, it is easy).*   - Collection of data on exogenous and occupational risk factors - HECSI - HEES - Quality of life assessment - Definition of etiological subtype(s) - Definition of morphological subtype(s) - Assessing whether positive patch tests reactions were relevant for CHE |
| --- |

CRF: Care Report Form; CHE: Chronic Hand Eczema; HECSI: Hand ECzema Severity Index; HEES Hand Eczema Extent Score
